# Genome-wide screening of copy number alterations and LOH events in renal cell carcinomas and integration with gene expression profile

**DOI:** 10.1186/1476-4598-7-6

**Published:** 2008-01-14

**Authors:** Ingrid Cifola, Roberta Spinelli, Luca Beltrame, Clelia Peano, Ester Fasoli, Stefano Ferrero, Silvano Bosari, Stefano Signorini, Francesco Rocco, Roberto Perego, Vanessa Proserpio, Francesca Raimondo, Paolo Mocarelli, Cristina Battaglia

**Affiliations:** 1 Dept. of Biomedical Sciences and Technologies and CISI, University of Milan, Milan, Italy; 2 Institute of Biomedical Technologies (ITB), National Research Council (CNR), Milan, Italy; 3 Pathological Anatomy Unit, Dept. of Medicine, Surgery and Dentistry, San Paolo Hospital, University of Milan, Milan, Italy; 4 Department of Laboratory Medicine, Desio Hospital, University of Milano-Bicocca, Milan, Italy; 5 Institute of Urology, IRCCS Policlinico, University of Milan, Milan, Italy; 6 Department of Experimental Medicine, University of Milano-Bicocca, Milan, Italy

## Abstract

**Background:**

Clear cell renal carcinoma (RCC) is the most common and invasive adult renal cancer. For the purpose of identifying RCC biomarkers, we investigated chromosomal regions and individual genes modulated in RCC pathology. We applied the dual strategy of assessing and integrating genomic and transcriptomic data, today considered the most effective approach for understanding genetic mechanisms of cancer and the most sensitive for identifying cancer-related genes.

**Results:**

We performed the first integrated analysis of DNA and RNA profiles of RCC samples using Affymetrix technology. Using 100K SNP mapping arrays, we assembled a genome-wide map of DNA copy number alterations and LOH areas. We thus confirmed the typical genetic signature of RCC but also identified other amplified regions (e.g. on chr. 4, 11, 12), deleted regions (chr. 1, 9, 22) and LOH areas (chr. 1, 2, 9, 13). Simultaneously, using HG-U133 Plus 2.0 arrays, we identified differentially expressed genes (DEGs) in tumor vs. normal samples. Combining genomic and transcriptomic data, we identified 71 DEGs in aberrant chromosomal regions and observed, in amplified regions, a predominance of up-regulated genes (27 of 37 DEGs) and a trend to clustering. Functional annotation of these genes revealed some already implicated in RCC pathology and other cancers, as well as others that may be novel tumor biomarkers.

**Conclusion:**

By combining genomic and transcriptomic profiles from a collection of RCC samples, we identified specific genomic regions with concordant alterations in DNA and RNA profiles and focused on regions with increased DNA copy number. Since the transcriptional modulation of up-regulated genes in amplified regions may be attributed to the genomic alterations characteristic of RCC, these genes may encode novel RCC biomarkers actively involved in tumor initiation and progression and useful in clinical applications.

## Background

Clear cell (or conventional) renal cell carcinoma (RCC) accounts for about 85% of all primary kidney malignancies and, although familial forms of RCC exist, the disease is more often sporadic[1]. This pathology is associated with genetic alterations affecting particular chromosomes [2,3]. The most frequent findings are deletions and unbalanced translocations involving chromosome 3p, resulting in the loss of specific regions, e.g. 3p25-p26 including the von Hippel-Lindau (*VHL*) gene locus [4]. Duplications of chromosomes 5q and 7 and deletions on chromosomes 6q, 8p, 9p and 14q are other typical chromosomal abnormalities. Another recurrent RCC genetic feature is a particular pattern of loss of heterozygosity (LOH), i.e. the change from a heterozygous genotype in a normal sample to a homozygous one in a tumor, with a high frequency of allelic imbalances on chromosome 3p in conjunction with 6q, 8p, 9p and q, and 14q [5]. These DNA alterations define a specific pattern of genetic instability, which represent a tumor-specific molecular fingerprint potentially useful for diagnostic applications [3]. In addition, some of these DNA alterations have been associated with tumor progression and metastatic potential, and so may be useful prognostic indicators [2]. Furthermore, many studies have profiled transcriptional patterns in RCC samples, but a univocally accepted gene expression signature is lacking [6].

Until now, chromosomal imbalances (i.e. amplifications and deletions) in neoplastic diseases have been studied with a variety of methods, such as cytogenetic techniques, comparative genomic hybridization (CGH) and the newest array-CGH, and fluorescence in situ hybridization (FISH), but each suffers limitations in resolution or high-throughput capacity [7,8]. The recent development of single nucleotide polymorphism (SNP) array technology has greatly improved the detection of DNA copy number (CN) changes, and now allows the simultaneous genotyping of more than 100,000 polymorphic loci distributed across all human chromosomes and thus the high-resolution scanning of the entire genome [9]. Moreover, by allelotyping the DNA sequence, this technology permits the detection of LOH events, which has principally been performed by investigating microsatellite markers at specific genomic regions. The assessment of areas rich in LOH events is a useful approach for identifying regions potentially harboring novel tumor suppressor genes (TSGs) [10]. Thus, SNP mapping technology permits the simultaneous analysis, at whole genome level and on the same platform, of chromosomal and allelic imbalances. Moreover, it permits the distinction between LOH associated with CN changes (such as hemizygous deletions) and that associated with a CN neutral status, due to different mechanisms including mitotic recombination, gene conversion and mitotic non-disjunction leading to uniparental disomy (UPD). Simultaneous assessment of LOH and CN changes at high resolution (more than 50,000 SNPs) has been recently performed in prostate cancers [11] and gliomas [12], but not yet in RCC.

There is an increasing tendency to combine genomic analysis with transcriptomic profiles, in order to study the relation between CN changes and gene expression levels. The integration of CGH profiles and transcriptional data has demonstrated that CN alterations have a clear impact on expression levels, in a variety of tumors [13-15]. Currently, this dual strategy is considered the most effective approach for interpreting genome-wide data about DNA and RNA anomalies in cancer, in order to identify chromosomal regions and genes involved in tumor initiation and progression. The proof of principle comes from the study by Garraway *et al*. [16], in which the combined analysis of genome-wide SNP-based CN data and expression profiles led to the identification of a candidate lineage-specific oncogene associated with a novel amplification in malignant melanoma. Concerning RCC, Furge *et al*. [17] developed a computational approach to predict cytogenetic abnormalities from gene expression data and confirmed alterations on chromosomes 3 and 5 as the typical DNA signature of this pathology. However, an integrated analysis of DNA and RNA from RCC samples using genome-wide approaches has not yet been reported.

In this study, we applied the 100K SNP mapping array technology to assemble a detailed genome-wide map of copy number alterations (CNAs) and LOH events in a collection of 27 RCC tissue samples. Moreover, we combined genomic data with the transcriptomic profile to identify chromosomal regions and individual genes modulated by the underlying DNA alterations and thus potentially useful in clinical applications as novel RCC constitutive markers.

## Results

### Visualization of the genomic profile of RCC samples

**Figure 1:**
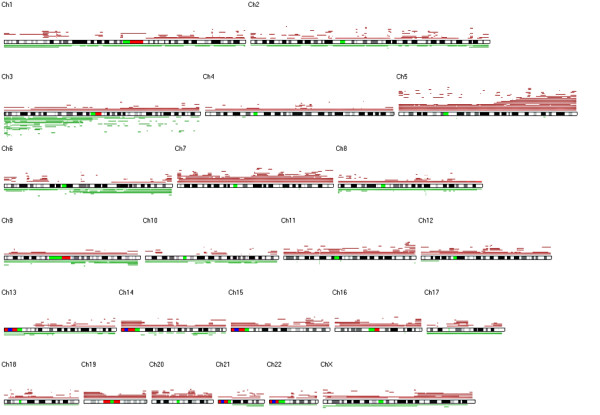
**DNA copy number alterations in the 27 RCC samples as identified by CNAG v2.0 software**. All chromosomes are represented with cytobands (black and white blocks), centromere (green blocks) and heterochromatic regions (red and blue blocks). Each tumor sample was compared to its matched normal blood sample, and regions of DNA copy number gain (red lines) and copy number loss (green lines) were plotted along each chromosome. Datasets from only GeneChip^® ^50K Hind arrays were used.

DNA copy number alterations (CNAs) were assessed at the genomic level in a set of 27 RCC samples, using GeneChip^® ^50K Hind array data and CNAG v2.0 software. Regions of CN gain and loss occurring in each sample along the entire genome are shown in a unique plot (Figure 1). All autosomes were affected by CN gain or loss, or both. The most recurrent CN gains were on chromosomes 5, 7, 11, 12, 14, 15, 16, 19 and 20, while CN losses mostly occurred on chr. 3p, 6q, 9q and 10q. Taken as a whole, these results illustrate a great heterogeneity in CNA profile among the 27 tumor samples.

To increase the resolution of our analysis, we combined data from both GeneChip^® ^50K Xba and 50K Hind arrays and performed a comprehensive analysis using dChip2006 software. For each chromosome, we assembled a detailed map showing both CNAs and LOH regions in each tumor sample (Additional file 1). By a visual inspection, each sample presented a specific pattern of CNAs, with different distributions and lengths, thus confirming the wide heterogeneity previously observed. Taking into account the whole sample set, the chromosomes harboring the largest aberrant regions were 3, 4, 5, 6, 7, 9, 12 and 20, while regarding LOH events, chromosomes 3, 6 and 9 were the most commonly affected. By simultaneously mapping both CNAs and LOH regions along all chromosomes, we observed that LOH events were usually accompanied by CN loss or CN neutral status, except for single small regions on chr. 2, 9, 11, 13, 14 and 16 showing LOH and concomitant CN gain. Thus, this analysis offers an immediate and comprehensive view of all CNAs and LOH events in the 27 tumor samples, allowing a more complete understanding of the complex genetic rearrangements underlying RCC pathology.

### Genome-wide assessment of LOH events in RCC samples

Using dChip2006 we identified chromosomal regions affected by significant LOH over all tumor samples. Calculating the probability across the whole sample set, we obtained LOH scores ranging from 0.07 to 0.22 and a corresponding number of affected chromosomes progressively decreasing from 15 to 1 (Table 1). Interestingly, setting LOH threshold at the maximum value of 0.22, only chromosome 3p was significantly affected by LOH, highlighting the high frequency and importance of this alteration in RCC pathology. To reach the best compromise between stringency and background noise, we selected regions with LOH score greater or equal to 0.15, thus identifying six chromosomes significantly affected by LOH over all samples, i.e. 1p, 2q, 3p, 3q, 6q, 9q and 13q.

**Table 1:** Chromosomes affected by significant LOH according to LOH score thresholds as calculated by dChip2006.

**LOH threshold**	**Chromosomes, n**	**Affected chromosomal arms**
0.07	15	1p, 1q, 2p, 2q, 3p, 3q, 5q, 6p, 6q, 8p, 8q, 9p, 9q, 10p, 10q, 12p, 12q, 13p,13q, 14q, 17p, 17q, 18p, 18q, 21q, 22q
0.11	11	1p, 1q, 2p, 2q, 3p, 3q, 6p, 6q, 8p, 8q, 9p, 9q, 10p, 10q, 12p, 13q, 17q, 21q
0.15	6	1p, 2q, 3p, 3q, 6q, 9q, 13q
0.19	2	3p, 6q
0.22	1	3p

Abbreviations: p, short arm; q, long arm.

To further investigate these significant LOH regions, we calculated their exact position, length and frequency (Table 2). We found 15 LOH regions distributed across the six chromosomes, among which four were located on chr. 3p and six were on chr. 6q. Chromosomes 1p, 2q, 9q and 13q had only one significant LOH region each. These regions spanned from 1 to 4 Mb, with frequencies ranging from 3 (11%) to 11 samples (41%). Interestingly, the most frequent LOH region was also one of the largest, covering 4 Mb on chr. 3p26.2-p25.3 and adjacent to other 4 Mb on chr. 3p24.3 and 1 Mb on chr. 3p14.3 showing LOH events in 26% and 30% samples, respectively, thus confirming the crucial involvement of chr. 3p in RCC etiology. Moreover, chromosome 6q presented three large LOH areas from 6q22.1 to 6q25.3, suggesting the presence of a wide region potentially involved in RCC pathology. Considering the corresponding DNA copy number status, these LOH regions were associated with either CN loss or CN neutral status, while no CN gain events were observed (Table 2). In particular, CN loss accompanied LOH on chr. 1p and 9q, while diploid copy number was retained on chr. 2q and 13q. Chromosomes 3 and 6 showed a more complex pattern, presenting LOH events with both CN loss and neutral in different tumor samples. Specifically, on chr. 3p, all LOH areas had no CN change, except for 3p26.2-p25.3, the most frequent and largest LOH region, which presented both CN loss and CN neutral status in 4 and 7 samples, respectively.

**Table 2:** Chromosomal regions affected by significant LOH in the 27 RCC samples.

Chromosome	Affected cytobands	Region start-end positions (Mb)	Total length of LOH region (Mb)	No. of samples with LOH events (%)	Copy number status	
					Loss	Neutral
					(no. of samples)	
1	p36.22-36.21	12–13	1	3 (11%)	2	1
2	q36.3	226–227	1	5 (18%)	1	4
3	p26.3	0–1	1	3 (11%)	0	3
	p26.2-p25.3	5–9	4	11 (41%)	4	7
	p24.3	15–22	4	7 (26%)	0	7
	p14.3	56–57	1	8 (30%)	0	8
	q22.1	132–133	1	5 (18%)	0	5
6	q16.1	95–96	1	4 (15%)	0	4
	q22.1-q22.31	115–119	3	5 (18%)	1	4
	q22.31	123–124	1	4 (15%)	3	1
	q22.33-q23.2	130–133	3	4 (15%)	1	3
	q25.2-q25.3	153–160	2	4 (15%)	3	1
	q26	163–164	1	4 (15%)	1	3
9	q31.1-q31.2	107–108	1	4 (15%)	4	0
13	q33.2	104–105	1	3 (11%)	0	3

Regions with LOH score greater than or equal to 0.15 (as calculated by dChip2006) were selected.

Abbreviations: p, short arm; q, long arm; Mb, megabase.

When we referred back to the individual samples, some interesting patterns emerged. Noticeably, on chr. 6q, 9q and 13q, we observed groups of samples showing similar LOH patterns, i.e. samples no. 27CG, 46SA and 60CC for LOH on chr. 6q, samples no. 33BV, 35PA, 36MMl on chr. 9q, and samples no. 36MMl, 46SA, and 60CC on chr. 13q (Additional file 1). The fact that these cases presented biological features of potential aggressiveness (relatively larger tumor diameter, and higher tumor stage and grade) or clinical evidence of invasive capacity (cases no. 35PA and 60CC had tumor recurrence) suggests that their similar LOH patterns on chr. 6q, 9q and 13q may be a specific fingerprint of RCC malignant potential. Among the six LOH regions on chr. 6q, four had diploid copy number in most samples, while two (on chr. 6q22.31 and 6q25.2-q25.3, respectively) were accompanied by CN loss in 3 of 4 cases. Interestingly, the three samples with CN loss were no. 27CG, 46SA and 60CC, suggesting the presence of a particular combined pattern of LOH and CN loss distinguishing these cases. Taken as a whole, these results allowed the assembly of a detailed map of significant LOH regions across the whole sample set and evidenced the presence of different mechanisms, such as hemizygous deletions and uniparental disomy (UPD) events, accompanying these allelic imbalances.

### Genome-wide assessment of CNAs in RCC samples

To have a comprehensive view of all DNA aberrations in RCC samples, we also assessed CNAs occurring in regions without significant LOH events (LOH score < 0.15) (Table 3). We found 17 regions with CN gain distributed across nine chromosomes. These amplifications occurred in at least 11 samples (41%) and had lengths ranging from 4 to 12 Mb. Chromosome 5 was the most affected, with a 10-Mb amplification on the p arm and three large regions covering the entire q arm. In addition, chromosome 7 presented three amplified regions on both p and q arms, chromosomes 4 and 11 showed CN gain events spanning over most of the p arm and part of the q arm, and chromosome 12 had CN gain on most of the q arm. Considering CN loss events, fewer chromosomes were affected and for smaller extents but the frequencies of affected samples were greater (Table 3). We identified six deleted regions, from 2 to 5 Mb in length, distributed across four chromosomes. The most recurrent deletions, observed in 26 samples (96%), occurred in the region from chr. 3p26.3 to 3p25.1. Moreover, at least 21 samples (78%) had CN loss on chr. 1p36.32-p36.11, 9q33.3-q34.11 and 22q13.1-q13.2. Overall, this analysis provided a comprehensive and detailed view of all CNAs in the 27 RCC samples and, while confirming a great sample heterogeneity, highlighted some recurrent aberrant regions potentially relevant for RCC etiology and worthy of further investigation.

**Table 3:** Chromosomal regions affected by DNA copy number alterations.

Chromosome	Affected cytobands	Region start-end positions (Mb)	Total length of aberrant regions (Mb)	No. of samples (%)
Regions of CN gain				
4	p15.1-q13.1	29–62	5	11 (41%)
	q26-q28.3	117–137	5	11 (41%)
5	p15.1-p14.1	17–29	10	11 (41%)
	q11.2-q12.3	53–66	5	11 (41%)
	q14.3-q21.3	84–105	12	11 (41%)
	q23.1-q34	119–163	11	11 (41%)
7	p21.3-p21.2	8–14	5	11 (41%)
	q21.11	81–85	4	11 (41%)
	q31.31-q31.32	118–122	4	11 (41%)
11	p15.1-q12.1	21–57	11	11 (41%)
	q14.2-q22.3	87–107	10	11 (41%)
12	q12-q15	35–69	10	11 (41%)
	q21.31-q23.1	82–100	5	11 (41%)
14	q21.1-q21.3	41–48	4	11 (41%)
16	p11.2-q11.1	34–39	5	11 (41%)
19	p12-p11	24–28	4	12 (44%)
22	p13-p11.2	0–9	9	12 (44%)
Regions of CN loss				
1	p36.32-p36.31	5–7	2	25 (93%)
	p36.11	24–26	2	21 (78%)
3	p26.3-p26.2	3–5	2	26 (96%)
	p25.3-p25.1	9–14	5	26 (96%)
9	q33.3-q34.11	128–130	2	21 (78%)
22	q13.1-q13.2	38–40	2	21 (78%)

None of these regions have concomitant significant LOH (score ≥ 0.15).

Abbreviations: CN, copy number; p, short arm; q, long arm; Mb, megabase.

The aberrant genomic regions listed in Tables 2 and 3 contain a total of 493 known genes (Additional file 2). Specifically, 194 genes mapped to CN gain regions distributed on chr. 4 (3 genes, among which the two protocadherins *PCDH7 *and *PCDH10*), chr. 5 (70 genes, e.g. the cadherin family genes, the two granzymes *GZMK *and *GZMA*, *CCNG1 *and *HMMR*), chr. 7 (21 genes, e.g. *HGF*), chr. 11 (56 genes, among which three caspases and *PDGFD*), chr. 12 (40 genes), chr. 14 (3 genes) and chr. 19 (1 gene). No genes were located in amplified regions on chr. 16 and 22. Similarly, we found 155 genes in CN loss regions distributed on chr. 1 (47 genes, e.g. IL-22 and IL-28 receptors), chr. 3 (52 genes, e.g. two IL-17 receptors, *VHL *and *TIMP4*), chr. 9 (32 genes) and chr. 22 (24 genes). Regarding significant LOH regions, 144 genes were identified, divided in 105 genes located in regions with concomitant CN loss and 39 genes in regions with no CN change. Regarding cellular localization, genes in CN gain regions preferentially encoded integral plasma membrane proteins, while genes mapping to CN loss regions and LOH regions encoded proteins located in cytoplasm and intracellular organelle membranes. All these genes may be of particular interest for RCC pathology, since among them could be oncogenes or TSGs located in amplified or deleted regions respectively, and thus potentially involved in tumor etiology.

### Transcriptomic profiling of RCC samples

Comparing 16 tumor to 11 normal cortical samples, we identified 2922 differentially expressed genes (DEGs), comprising 1511 up-regulated and 1411 down-regulated genes (Additional file 3). Functional annotation analysis revealed that up-regulated genes mostly belonged to the classes cell cycle (e.g. *PCNA*, *RB1*, *BRCA1*, *RGS1*, *RGS5*, *CA9*, five cyclins and three caspases), focal adhesion (including *VEGF*, *CAV1*, *CAV2*, *VIM*, *PDGFD*, five integrins, seven collagen genes) and extracellular matrix-interaction (e.g. *CXCR4*, four laminins and three cadherins), as well as to Toll-like receptor and T-cell receptor signaling pathways (including TNF and IFN ligands and receptors, six interleukin receptors, four granzyme genes). Differently, down-regulated genes were principally related to aminoacid and fatty acid metabolic pathways (e.g. *AGMAT*, four *ADH *genes, six *ALDH *genes and nine *CYP450 *members) and to glycolysis and gluconeogenesis (e.g. *G6PC*, *ALDOB*, *FBP1*). Considering chromosomal locations, up-regulated genes were principally located on chromosomes 5 (138 genes), 11 (106 genes), 12 (102 genes), 7 (90 genes), 4 (75 genes) and 15 (60 genes), while chromosomes 1 (174 genes), 3 (124 genes), 17 (97 genes), 9 (78 genes) and 14 (56 genes) were enriched in down-regulated genes.

When our 2922 DEGs were matched to the 294 DEGs that are commonly detected by the two studies of Jones *et al*. and Lenburg *et al*. [18], similar findings were obtained regarding 201 genes, comprising 125 up-regulated (e.g. *RGS1*, *RGS5*, *CXCR4*, *LOX*, *VEGF*, *CAV1*, *CAV2*, *HIG2*, *CA9*) and 76 down-regulated genes (e.g., *EGF*, *CHL1*, *DEFB1*, *CALB1*, *SERPINA5*) (Additional file 3). Since these genes have been found to be differentially expressed by three separate studies, they are of particular interest, since among them could be novel markers involved in RCC oncogenesis.

### Integration of copy number data with transcriptomic profile

To search for correlations between DNA alterations and expression profile, the list of genes located in aberrant regions was matched with DEG list, thus identifying 71 differential genes mapping in aberrant regions (Additional file 4). Specifically, among the 37 DEGs in CN gain regions, 27 (73%) were up-regulated in tumor samples and, of the 27 DEGs in CN loss regions (with and without concomitant LOH), 14 (52%) were down-regulated. Moreover, 7 genes mapped to LOH regions with CN neutral status and all had reduced transcriptional levels. These results illustrate the clear impact of CNAs on gene expression modulation.

**Figure 2:**
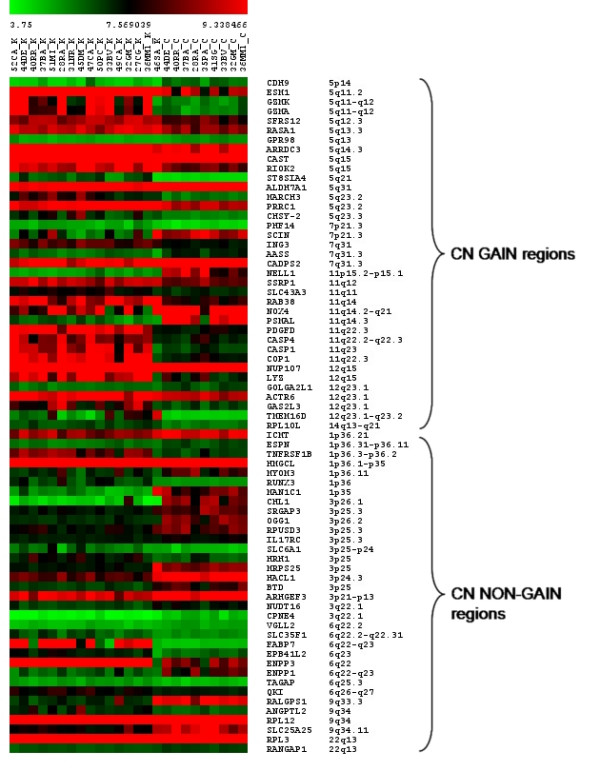
**TMEV heat map showing differentially expressed genes (DEGs) mapping in aberrant chromosomal regions**. DEGs calculated by SAM analysis and located in regions affected by significant amplification (CN gain regions) or other DNA alterations, such as deletion or LOH events (CN non-gain regions) were selected. For each tumor sample (K) and normal control (C), raw signal intensities converted into expression values by RMA procedure were visualized using a color gradient from green to red. For each gene (in row), gene symbol and corresponding cytoband are reported. Abbreviations: p, short arm; q, long arm.

To further investigate the relation between genomic position and transcriptional regulation, we assembled a heat map showing the expression levels of the 71 DEGs plotted in order of their chromosomal position within aberrant regions (Figure 2). Interestingly, despite a wide sample heterogeneity, the groups of tumor and normal samples were easily distinguishable. Moreover, up-regulated DEGs were often grouped within amplified regions to form clusters of concordantly modulated genes, as on chr. 5 (12 genes from 5q11.2 to 5q23.3, e.g. *ESM1*, *GZMK *and *GZMA*), chr. 11 (7 genes from 11q11 to 11q22.3, including *PDGFD *and *CASP1*) and chr. 12 (5 genes from 12q15 to 12q23.1). This distribution highlights the strong relation between regional DNA amplification and increased transcriptional level of the corresponding genes. Differently, in non-gain regions this clusterization of DEGs was less marked. Exclusively on chr. 3, 11 of the 13 DEGs were down-regulated and formed a large transcriptionally reduced region spanning the entire chromosome. Here, genes in LOH areas with CN neutral were also down-regulated, supporting the crucial importance in RCC pathology of chr. 3 inhibition by various genomic mechanisms. Altogether, these results demonstrate a striking association between DNA alteration profile and gene expression levels. Moreover, they evidence a stronger influence on transcriptional modulation by CN gain than CN loss events. Thus, amplified regions identified in this analysis are of particular importance since they may contain genes which, being up-regulated due to underlying DNA amplification events, could be novel RCC constitutive markers.

## Discussion

In this study, we used the 100K SNP mapping array technology to investigate DNA copy number alterations and LOH events in a collection of 27 RCC tissue samples. Simultaneously, transcriptomic analysis was performed to identify differentially expressed genes potentially involved in tumor etiology. To narrow down the number of candidate genes to those whose altered expression level was attributable to underlying DNA alterations, genomic and transcriptomic data were combined at genome level. This permitted us to identify specific chromosomal regions with concordant alterations in DNA and RNA profiles. These regions are of particular interest since they contain genes which, being transcriptionally modulated due to genomic alterations, could be novel RCC constitutive markers actively involved in tumor initiation and progression and useful in clinical applications.

To our knowledge, this is the first study assembling a genome-wide map of CNAs and LOH events characterizing RCC pathology. We documented CN loss regions on chromosomes 3p and 6q, thus confirming the genetic signature typical of RCC while greatly improving the resolution compared to previous studies employing cytogenetic and CGH techniques [2,3,19]. We also observed novel CN loss regions on chr. 1p, 9q and 22q. We confirmed the presence of genomic regions affected by LOH on chromosomes 3p, 6q and 9q, as previously identified by Thrash-Bingham *et al*. in a microsatellite analysis on all chromosomes [5]. In addition, our data revealed novel LOH sites on chromosomes 1p, 2q, 3q and 13q. Taking into account the DNA copy number status, we distinguished LOH events associated with CN loss (e.g. on chromosomes 1p and 9q) highlighting the presence of hemizygous deletions, and LOH events with CN neutral status (e.g. on chromosomes 2q and 13q) indicating the occurrence of concomitant genetic mechanisms (including mitotic recombination, gene conversion or UPD events) to maintain the normal diploidy status. It is widely accepted that regions of LOH accompanied by deletion (representing the second hit of Knudson's hypothesis) are of particular interest since they may contain TSGs actively involved in tumor etiology and thus completely inactivated [10]. Interestingly, we identified a novel LOH and CN loss site on chromosome 1p36.22-p36.21, a region frequently deleted also in glioma and neuroblastoma [20] and harboring potential TSGs, such as *IIp45 *(invasion inhibitory protein 45), found down-regulated in glioblastomas [21], and *DHRS3*, a dehydrogenase/reductase enzyme frequently inactivated by deletion in human neuroblastoma cell lines [22] and by promoter methylation in melanoma cell lines [23].

It is known that RCC is characterized by a particular genetic signature on chr. 3p [24]. We observed deletions affecting chr. 3p26.3-p26.2 and 3p25.3-p25.1 in 96% samples, thus confirming the crucial importance of inactivation of these regions in RCC oncogenesis [2]. Moreover, in 30% of samples we observed LOH with CN neutral status on chr. 3p14.3, a region already reported to be frequently affected by LOH in RCC pathology and harboring the *FHIT *gene, thought to be involved in tumor suppression in many neoplasms [25]. Differently, the most frequent LOH area, mapped on chr. 3p26.2-p25.3 and observed in 41% samples, showed a more complex DNA arrangement, presenting both CN loss and neutral status in different tumor samples. This region harbors in 3p25.3 the *VHL *gene, the main TSG implicated in the early steps of RCC oncogenesis[4]. As previously reported, in 50–70% of RCC sporadic forms, *VHL *gene is completely inactivated through a combination of various mechanisms such as mutations, promoter methylation, LOH and deletions [4]. VHL protein is a key regulator of the hypoxic response and controls the transcription of several genes. Concordantly with a VHL loss of function, we found increased expression of three known hypoxia-inducible genes: the carbonic anhydrase *CA9*, a cell growth regulator; the chemokine receptor *CXCR4*, which controls metastatic spread; and the vascular endothelial growth factor (*VEGF*), involved in cell adhesion and angiogenesis. These genes have already been reported to be up-regulated in RCC and other human cancers [26].

Combining our transcriptomic data with the publicly available dataset of Jones *et al*[18], we found a common gene signature comprising 201 differential genes. In addition, we found DEGs already reported as transcriptionally modulated in RCC samples by previous studies (Table 4).

**Table 4:** Selection of differentially expressed genes potentially involved in RCC pathology.

**Gene symbol (chr)**	**Gene name**	**Biological function**	**Differential expression in RCC**	**References**
*RGS5 *(1q)	Regulator of G-protein signalling 5	angiogenesis	up	[[18,27]]
*CXCR4 *(2q)	Chemokine C-X-C receptor 4	metastatic spread	up	[[18,26]]
*LOX *(5q)	Lysyl oxidase	metastatic spread	up	[[18,27]]
*VEGFA *(6p)	Vascular endothelial growth factor A	angiogenesis	up	[[18,26]]
*CAV1 *(7q)	Caveolin 1	cell adhesion and extracellular matrix interaction	up	[[18,41]]
*HIG2 *(7q)	Hypoxia-inducible protein 2	cell growth promotion	up	[[18,27,42]]
*CA9 *(9p)	Carbonic anhydrase IX	cell growth promotion	up	[[18,43]]
*VIM *(10p)	Vimentin	cell adhesion and extracellular matrix interaction	up	[44]
*NNMT *(11q)	Nicotinamide N-methyltransferase	methyltransferase activity	up	[[18,45]]
*EGLN3 *(14q)	Egl nine homolog 3	cell growth regulation	up	[[18,27]]
*DEFB1 *(8p)	Defensin beta 1	antimicrobial activity	down	[[44]]
*PVALB *(22q)	Parvalbumin	calcium ion binding	down	[[44]]

Differentially expressed genes were calculated by SAM analysis comparing 16 RCC tissue samples to 11 normal cortical tissues. Genes were selected by comparing our list with the RCC dataset of Jones *et al*. and with published literature. References specifically reporting a potential role of these genes in RCC pathology are indicated.

Although our analysis is limited by its relatively small sample collection compared to that of Zhao *et al*. [27], this is the first study to perform a genome-wide CNAs and LOH mapping using RCC and matched normal DNA from the same patients, together with gene profiling analysis. We documented a strong association between DNA alterations and gene expression levels and observed that CN gain events have a stronger influence on transcriptional modulation than CN loss events, in agreement with previous studies on other tumor types [28]. That gene amplification greatly enhances expression levels is supported by ample evidence from mammalian cell lines and tumors [29]. On the contrary, there are many aneuploidy-independent mechanisms leading to transcriptional down-modulation (e.g. mutation, promoter hypermethylation) [30]. Thus, we suggest that the recently developed approaches to infer "functional aneuploidy" of tumor samples by their transcriptomic profile [17,31] should take into account these differences.

Focusing on regions with concomitant CN gain events and up-regulated expression levels, we observed that over-expressed genes often mapped close to one another to form blocks of concordantly regulated genes. These regions may contain novel RCC constitutive markers, i.e. genes with up-regulated expression levels due to DNA amplification events particular of RCC histotype and necessary for its malignant transformation or progression. Up-regulated genes whose protein products are released to the extracellular space are ideal tumor markers for clinical applications, since they are detectable in patients' biological fluids without invasive procedures. Interestingly, within the amplified region on chromosome 5q11-q12, we found strongly increased expression levels of two adjacent genes encoding secreted proteins. *ESM1 *(endothelial cell-specific molecule 1, alias endocan), crucially involved in cell growth regulation, vascular remodeling and angiogenesis, is dramatically over-expressed in endothelial cells of kidney cancers and other tumors, in association with increased tumor aggressiveness and vascularity [32,33]. Moreover, elevated protein levels have been observed in serum of late-stage lung cancer patients, correlating inversely with both survival and time to tumor progression [34,35]. Next, *GZMA *gene, encoding a secreted granzyme involved in cytotoxic T cell-mediated immune response, is over-expressed in RCC tissues with high contents of natural killer cells and tumor-infiltrating lymphocytes [36]. Furthermore, in the amplified region on chromosome 11q22.3-q23, we found four adjacent over-expressed genes, including *PDGFD*, which encodes a secreted activator of the inflammatory response playing an important role in angiogenesis and is over-expressed in prostate carcinoma [37] and many tumor, including RCC, cell lines [38], and *CASP1*, which is up-regulated in pancreatic adenocarcinomas [39] and presents increased plasma levels in metastatic melanoma patients [40]. These genes and their encoded proteins will be further studied to determine if they have roles in RCC pathology and if they could be novel RCC biomarkers detectable in clinical biological fluids.

## Conclusion

By combining genomic and transcriptomic profiles from a collection of RCC samples, we identified at high resolution specific genomic regions concomitantly affected by DNA alterations and modulated expression levels. This study lays the groundwork for a more detailed search for novel RCC biomarkers associated with these aberrant regions, in particular in areas with increased DNA copy number. The integration of data from both DNA and RNA profiles should greatly improve the specificity of analysis and increase the possibility of identifying the genetic causes of tumor pathologies. Moreover, data from the follow-up evaluations of these RCC patients should enable us to validate this novel genomic RCC fingerprint.

## Methods

### Collection of RCC tissue samples and DNA/RNA extraction

A total of 30 patients with a diagnosis of clear cell renal carcinoma (RCC) were scheduled for surgical removal of kidney at San Paolo Hospital (University of Milan, Italy). All patients provided informed consent for the use of kidney tissues and blood samples in this multi-center research project, which was carried out according to the Declaration of Helsinki (as revised in 2004) and was approved by the ethic committee of San Paolo Hospital. Prior to surgery, a whole blood sample was collected from each case and stored at -20°C. Immediately after surgical removal of the kidney, a pathologist excised bioptic samples from both the tumor and the normal cortical areas. Fresh tissue samples were immersed in RNA *later *solution (Qiagen, Hilden, Germany) and stored at -80°C. Tumor masses were measured for longest diameter and scored for stage and nuclear grade according to the UICC pTNM staging system and Fuhrman's grading system, respectively.

Genomic DNA was extracted from tumor and blood samples using standard proteinase K-cell lysis and phenol/chloroform procedures. DNA was quantified by ND-1000 spectrophotometer (NanoDrop Technologies, Wilmington, DE) and stored at 4°C. Total RNA was extracted from tumor and cortical samples using RNeasy Mini extraction kit (Qiagen). Samples were quantified by spectrophotometry and assessed for quality by microcapillary electrophoresis on 2100 Bioanalyzer (Agilent Technologies, Palo Alto, CA). Total RNA was considered suitable for microarray analysis if the 28S/18S rRNA ratio was greater than 1.3. RNA samples were stored at -80°C until use. Overall, we obtained good-quality DNA from 27 tumor tissues and matched blood samples, and good-quality RNA from 16 tumor tissues and 11 healthy cortical samples (listed in Additional file 5).

### Sample preparation for Affymetrix GeneChip^® ^analysis

Genomic DNA from the 27 tumor tissues and corresponding blood samples was prepared for whole-genome SNP mapping using both GeneChip^® ^Human Mapping 50K Xba and 50K Hind assay kits (Affymetrix, Santa Clara, CA), according to the manufacturer's protocols. The combination of the two GeneChip^® ^Human Mapping arrays, referred to as 100K SNP mapping array set, allowed the genotyping of 116204 SNPs distributed over all chromosomes, excluding the Y chromosome. Total RNA from 16 tumor and 11 cortical tissues was prepared using GeneChip^® ^Two-Cycle Target Labeling assay kit (Affymetrix), according to the manufacturer's protocols, and hybridized onto GeneChip^® ^HG-U133 Plus 2.0 arrays (for complete information about methods see Additional file 6). All GeneChip^® ^data files are available at ArrayExpress repository (E-TABM-282, E-TABM-283 and E-TABM-284).

### Assessment of genomic CNAs and LOH events in RCC samples

Genomic regions with CNAs were first assessed using Copy Number Analyzer for GeneChip software (CNAG, v2.0). We compared the 27 tumor samples to the corresponding blood samples and displayed in a unique plot all CNAs occurring along each chromosome (for further information see Additional file 6). Datasets from only GeneChip^® ^50K Hind arrays were used. CNAs were also assessed using dChip2006 software, which offers the possibility of combining the two datasets from GeneChip^® ^50K Xba and 50K Hind arrays. dChip2006 was used to calculate a raw CN value for each SNP in tumor and normal samples. Then, we implemented a home-made procedure to calculate aberrant regions (for complete description of the procedure see Additional file 6). Moreover, dChip2006 was used to identify LOH events and to define a summary LOH score for each SNP taking into account all the 27 sample pairs. Then, applying a home-made procedure, we calculated regions affected by statistically significant LOH (for details see Additional file 6). In addition, the CN status of these regions was evaluated to distinguish LOH events with and without CN changes.Using the UCSC database, all genes located in aberrant regions were identified. Literature mining was carried out with MILANO tool, using specific cancer-related keywords.

### Transcriptomic profiling and integration with copy number data

Differential expression profiling was performed by SAM analysis comparing the 16 tumor to the 11 normal cortical samples to identify differentially expressed genes (DEGs). Functional annotation of the DEG list was carried out using DAVID database (for details about procedures see Additional file 6). Furthermore, we compared our DEG list with the RCC microarray expression dataset published by Jones *et al*. and including differential genes that overlap with the dataset provided by Lenburg *et al*. [18]. The DEGs found in common were functionally annotated with DAVID.

Finally, the list of genes in chromosomal regions with significant CNAs was compared to our DEG list to investigate the expression levels of genes in aberrant regions. For all genes found in common, expression values calculated for each tumor and normal sample were plotted in a heat map using the TIGR Multiple Experiment Viewer (TMEV, v4.0) (for details, Additional file 6).

## Competing interests

The author(s) declare that they have no competing interests.

## Authors' contributions

IC and CP carried out microarray experiments. RS and LB performed statistical and bioinformatical analyses. EF and SS prepared DNA and RNA samples. SF and SB carried out histological classification. FR coordinated patient enrollment and surgery. VP contributed to clinical database management. PM coordinated the research and the clinical follow-up of patients. CB supervised the study and, together with IC, wrote the manuscript. CB and PM are guarantors of the study. All authors read and approved the final manuscript.

## Supplementary Material

Additional file 1 **Chromosomal maps of LOH regions and DNA copy number alterations in the 27 RCC samples**. Each map represents one single chromosome (from 1 to 22). For each tumor sample compared to corresponding normal control, probability of LOH events as calculated by dChip2006 was displayed along the entire chromosome, from p to q arm, according to the following color-coded legend: blue, 1; yellow, 0.5; white, 0. Similarly, inferred copy number values calculated by dChip2006 were plotted using a color scale (from white to red) to indicate increasing copy number value. Abbreviations: p, short arm; q, long arm. Click here for file

Additional file 2 **Genes mapping in regions harbouring recurrent DNA copy number alterations or significant LOH events**. Genes are listed in order of chromosome and genomic start position. Using UCSC genome browser a complete gene annotation was retrieved including Entrez Gene ID, Gene symbol, Gene name, chromosome, cytoband and genomic start and end positions. In addition, each gene is flagged by a label (CN gain, CN loss, LOH+CN loss, LOH+CN neutral) indicating the type of DNA alteration occurring in the corresponding region. Click here for file

Additional file 3 **Complete list of differentially expressed genes (DEGs) in RCC tissues as compared to normal cortical samples**. GeneChip^® ^HG-U133 Plus 2.0 array probesets were annotated using an updated custom chip description file and metaprobes corresponding to unique Entrez Gene IDs having differential expression levels in tumor samples as compared to normal samples were calculated by the t statistic of the significance analysis of microarrays (SAM). For each listed gene, corresponding t score and fold change calculated by SAM analysis are reported, in addition to a flag (up/down) indicating the expression level in our RCC sample set. Furthermore, the last two columns indicate DEGs and corresponding fold change (FC) found in common between the two studies of Jones *et al*. and Lenburg *et al*. Click here for file

Additional file 4 **Differentially expressed genes (DEGs) mapping in aberrant chromosomal regions**. For each gene (reported as Entrez Gene ID, Gene symbol and Gene name), genomic position (in terms of chromosome, cytoband and start and end positions) and type of DNA alteration (labelled as CN gain, CN loss, LOH+CN loss and LOH+CN neutral) occurring in the corresponding region are shown, in addition to the fold change (FC) value calculated by SAM analysis. "CNA/FC concordance" flag indicates the concordant direction between DNA aberration type and differential gene expression level, i.e. the concomitant occurrence of DNA gain events and up-regulated transcriptional levels (concordant UP) or of DNA loss events and down-modulated transcriptional levels (concordant DOWN) of genes located within. Lastly, for each tumor sample (K) and normal control (C), array fluorescent signal intensities of each listed gene converted into RMA expression values are shown. Click here for file

Additional file 5 **Clear cell renal carcinoma samples analyzed in this study and corresponding clinical data**. Cases are listed in order of increasing tumor size, stage and grade classes. For each case, patient's gender, tumor size at the time of surgery, tumor stage and nuclear grade classifications and time elapsed from surgery are reported. These samples were analyzed for genomic (on GeneChip 100K SNP mapping arrays) or transcriptomic (on HG-U133 Plus 2.0 arrays) profiles. *All patients were scheduled for total removal of affected kidney, except for two which underwent partial nephrectomy. **Two patients presented tumor recurrence. Abbreviations: M, male; F, female; NA, data not available. Click here for file

Additional file 6 Complete description about methods used to prepare biological samples for microarray experiments and about statistical procedures implemented to analyse data. Click here for file
