# Supplementary material for: Genome-wide screening of copy number alterations and LOH events in renal cell carcinomas and integration with gene expression profile
Source: Mol Cancer. 2008 Jan 14;7:6. doi: 10.1186/1476-4598-7-6 (PMC2253555; doi:10.1186/1476-4598-7-6)
Supplement: Additional file 1 — Chromosomal maps of LOH regions and DNA copy number alterations in the 27 RCC samples. Each map represents one single chromosome (from 1 to 22). For each tumor sample compared to corresponding normal control, probability of LOH events as calculated by dChip2006 was displayed along the entire chromosome, from p to q arm, according to the following color-coded legend: blue, 1; yellow, 0.5; white, 0. Similarly, inferred copy number values calculated by dChip2006 were plotted using a color scale (from white to red) to indicate increasing copy number value. Abbreviations: p, short arm; q, long arm. [file 1476-4598-7-6-S1.pdf]

Chro 1
Inferred Copy number
Proportional

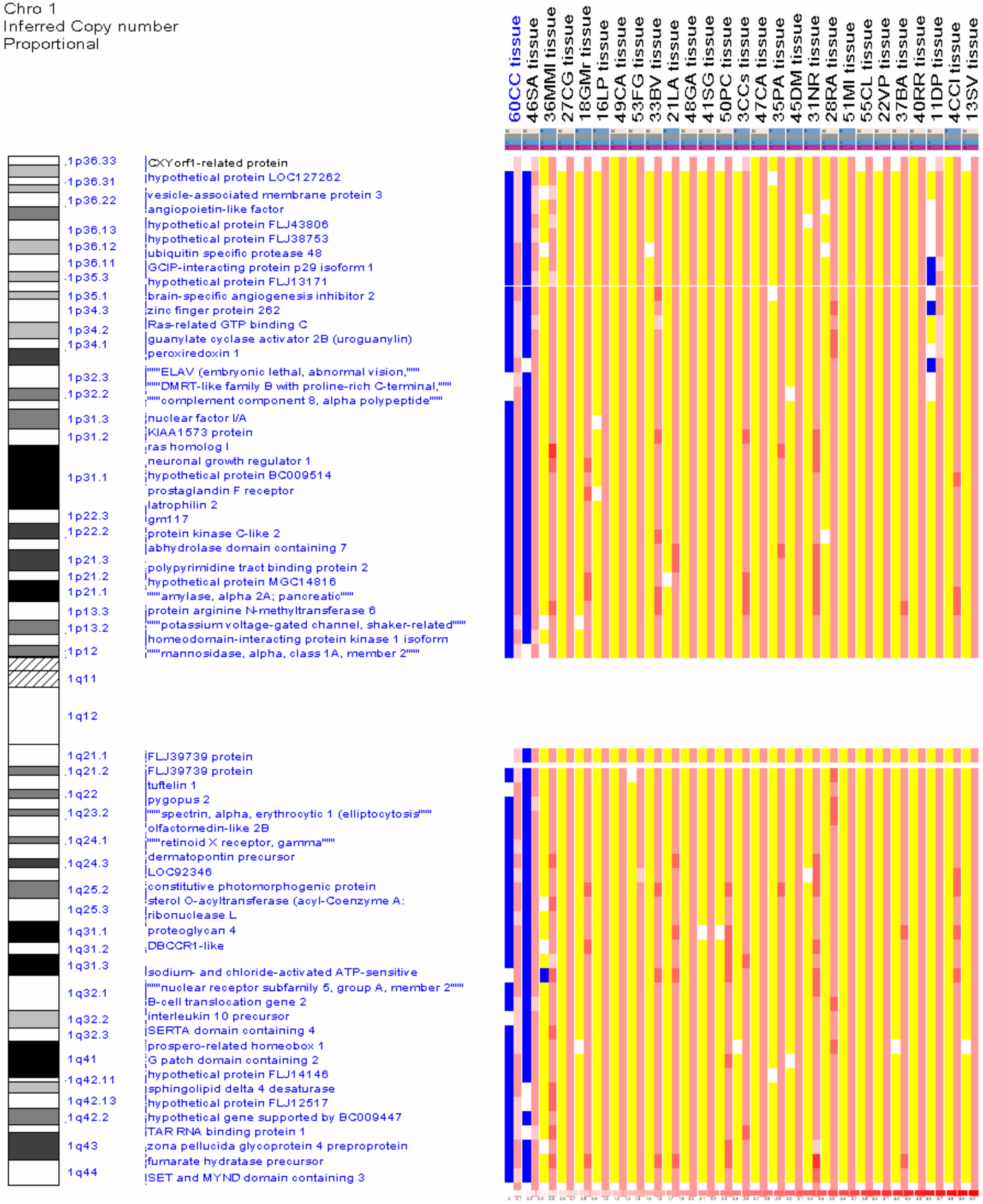

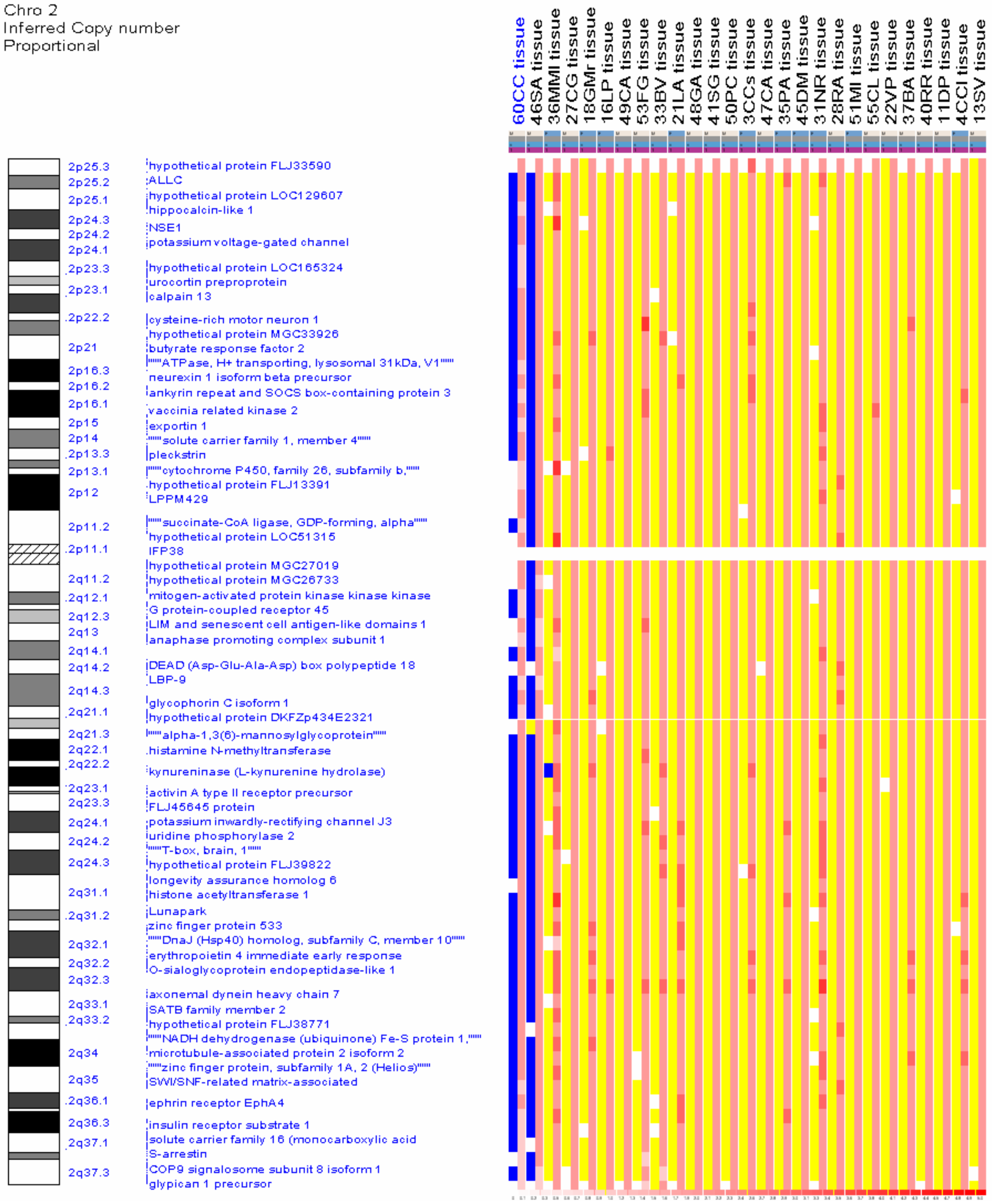

Chro 2
Inferred Copy number
Proportional
60CC tissue
46SA tissue
36MMI tissue
27CG tissue
18GMr tissue
16LP tissue
49CA tissue
53FG tissue
33BV tissue
21LA tissue
48GA tissue
41SG tissue
50PC tissue
3CCs tissue
47CA tissue
35PA tissue
45DM tissue
31NR tissue
28RA tissue
51MI tissue
55CL tissue
22VP tissue
37BA tissue
40RR tissue
11DP tissue
4CCI tissue
13SV tissue
2p25.3
2p25.2
2p25.1
2p24.3
2p24.2
2p24.1
2p23.3
2p23.1
2p22.2
2p21
2p16.3
2p16.2
2p16.1
2p15
2p14
2p13.3
2p13.1
2p12
2p11.2
2p11.1
2q11.2
2q12.1
2q12.3
2q13
2q14.1
2q14.2
2q14.3
2q21.1
2q21.3
2q22.1
2q22.2
2q23.1
2q23.3
2q24.1
2q24.2
2q24.3
2q31.1
2q31.2
2q32.1
2q32.2
2q32.3
2q33.1
2q33.2
2q34
2q35
2q36.1
2q36.3
2q37.1
2q37.3
hypothetical protein FLJ33590
ALLC
hypothetical protein LOC129607
hippocalcin-like 1
NSE1
potassium voltage-gated channel
hypothetical protein LOC165324
urocortin preproprotein
calpain 13
cysteine-rich motor neuron 1
hypothetical protein MGC33926
butyrate response factor 2
"""ATPase, H+ transporting, lysosomal 31kDa, V1"""
neurexin 1 isoform beta precursor
ankyrin repeat and SOCS box-containing protein 3
vaccinia related kinase 2
exportin 1
"""solute carrier family 1, member 4"""
pleckstrin
"""cytochrome P450, family 26, subfamily b,"""
hypothetical protein FLJ13391
LPPM429
"""succinate-CoA ligase, GDP-forming, alpha"""
hypothetical protein LOC51315
IFP38
hypothetical protein MGC27019
hypothetical protein MGC26733
mitogen-activated protein kinase kinase kinase
G protein-coupled receptor 45
LIM and senescent cell antigen-like domains 1
anaphase promoting complex subunit 1
DEAD (Asp-Glu-Ala-Asp) box polypeptide 18
LBP-9
glycophorin C isoform 1
hypothetical protein DKFZp434E2321
"""alpha-1,3(6)-mannosylglycoprotein"""
histamine N-methyltransferase
kynureninase (L-kynurenine hydrolase)
activin A type II receptor precursor
FLJ45645 protein
potassium inwardly-rectifying channel J3
uridine phosphorylase 2
"""T-box, brain, 1"""
hypothetical protein FLJ39822
longevity assurance homolog 6
histone acetyltransferase 1
Lunapark
zinc finger protein 533
"""DnaJ (Hsp40) homolog, subfamily C, member 10"""
erythropoietin 4 immediate early response
O-sialoglycoprotein endopeptidase-like 1
axonemal dynein heavy chain 7
SATB family member 2
hypothetical protein FLJ38771
"""NADH dehydrogenase (ubiquinone) Fe-S protein 1,"""
microtubule-associated protein 2 isoform 2
"""zinc finger protein, subfamily 1A, 2 (Helios)"""
SWI/SNF-related matrix-associated
ephrin receptor EphA4
insulin receptor substrate 1
solute carrier family 16 (monocarboxylic acid
S-arrestin
COP9 signalosome subunit 8 isoform 1
glypican 1 precursor

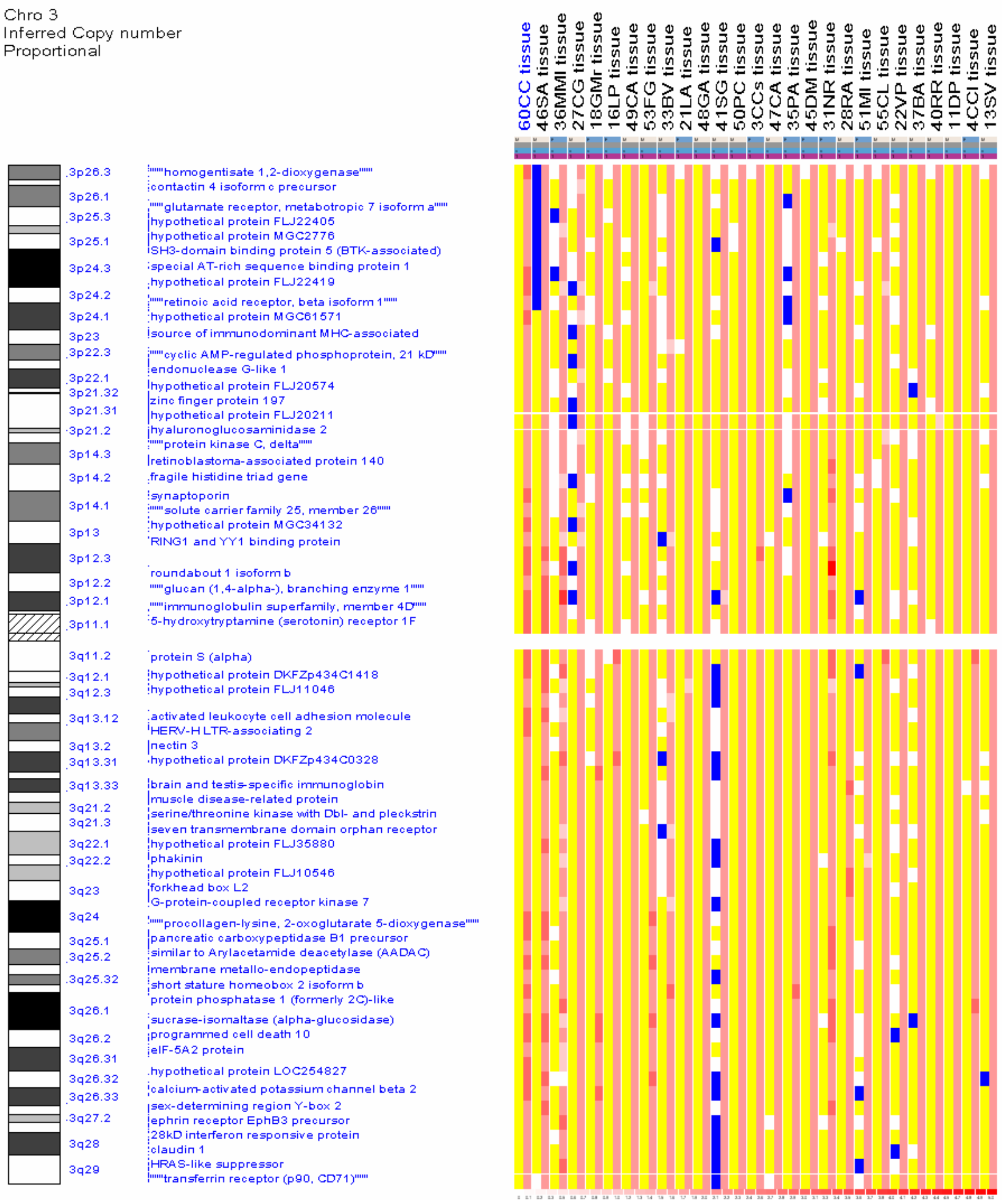
Chro 3
Inferred Copy number
Proportional
60CC tissue
46SA tissue
36MMI tissue
27CG tissue
18GMr tissue
16LP tissue
49CA tissue
53FG tissue
33BV tissue
21LA tissue
48GA tissue
41SG tissue
50PC tissue
3CCs tissue
47CA tissue
35PA tissue
45DM tissue
31NR tissue
28RA tissue
51MI tissue
55CL tissue
22VP tissue
37BA tissue
40RR tissue
11DP tissue
4CCI tissue
13SV tissue
3p26.3
3p26.1
3p25.3
3p25.1
3p24.3
3p24.2
3p24.1
3p23
3p22.3
3p22.1
3p21.32
3p21.31
3p21.2
3p14.3
3p14.2
3p14.1
3p13
3p12.3
3p12.2
3p12.1
3p11.1
3q11.2
3q12.1
3q12.3
3q13.12
3q13.2
3q13.31
3q13.33
3q21.2
3q21.3
3q22.1
3q22.2
3q23
3q24
3q25.1
3q25.2
3q25.32
3q26.1
3q26.2
3q26.31
3q26.32
3q26.33
3q27.2
3q28
3q29
"""homogentisate 1,2-dioxygenase"""
contactin 4 isoform c precursor
"""glutamate receptor, metabotropic 7 isoform a"""
hypothetical protein FLJ22405
hypothetical protein MGC2776
SH3-domain binding protein 5 (BTK-associated)
special AT-rich sequence binding protein 1
hypothetical protein FLJ22419
"""retinoic acid receptor, beta isoform 1"""
hypothetical protein MGC61571
source of immunodominant MHC-associated
"""cyclic AMP-regulated phosphoprotein, 21 kD"""
endonuclease G-like 1
hypothetical protein FLJ20574
zinc finger protein 197
hypothetical protein FLJ20211
hyaluronoglucosaminidase 2
"""protein kinase C, delta"""
retinoblastoma-associated protein 140
fragile histidine triad gene
synaptoporin
"""solute carrier family 25, member 26"""
hypothetical protein MGC34132
RING1 and YY1 binding protein
roundabout 1 isoform b
"""glucan (1,4-alpha-), branching enzyme 1"""
"""immunoglobulin superfamily, member 4D"""
5-hydroxytryptamine (serotonin) receptor 1F
protein S (alpha)
hypothetical protein DKFZp434C1418
hypothetical protein FLJ11046
activated leukocyte cell adhesion molecule
HERV-H LTR-associating 2
nectin 3
hypothetical protein DKFZp434C0328
brain and testis-specific immunoglobin
muscle disease-related protein
serine/threonine kinase with Dbl- and pleckstrin
seven transmembrane domain orphan receptor
hypothetical protein FLJ35880
phakinin
hypothetical protein FLJ10546
forkhead box L2
G-protein-coupled receptor kinase 7
"""procollagen-lysine, 2-oxoglutarate 5-dioxygenase"""
pancreatic carboxypeptidase B1 precursor
similar to Arylacetamide deacetylase (AADAC)
membrane metallo-endopeptidase
short stature homeobox 2 isoform b
protein phosphatase 1 (formerly 2C)-like
sucrase-isomaltase (alpha-glucosidase)
programmed cell death 10
eIF-5A2 protein
hypothetical protein LOC254827
calcium-activated potassium channel beta 2
sex-determining region Y-box 2
ephrin receptor EphB3 precursor
28kD interferon responsive protein
claudin 1
HRAS-like suppressor
"""transferrin receptor (p90, CD71)"""

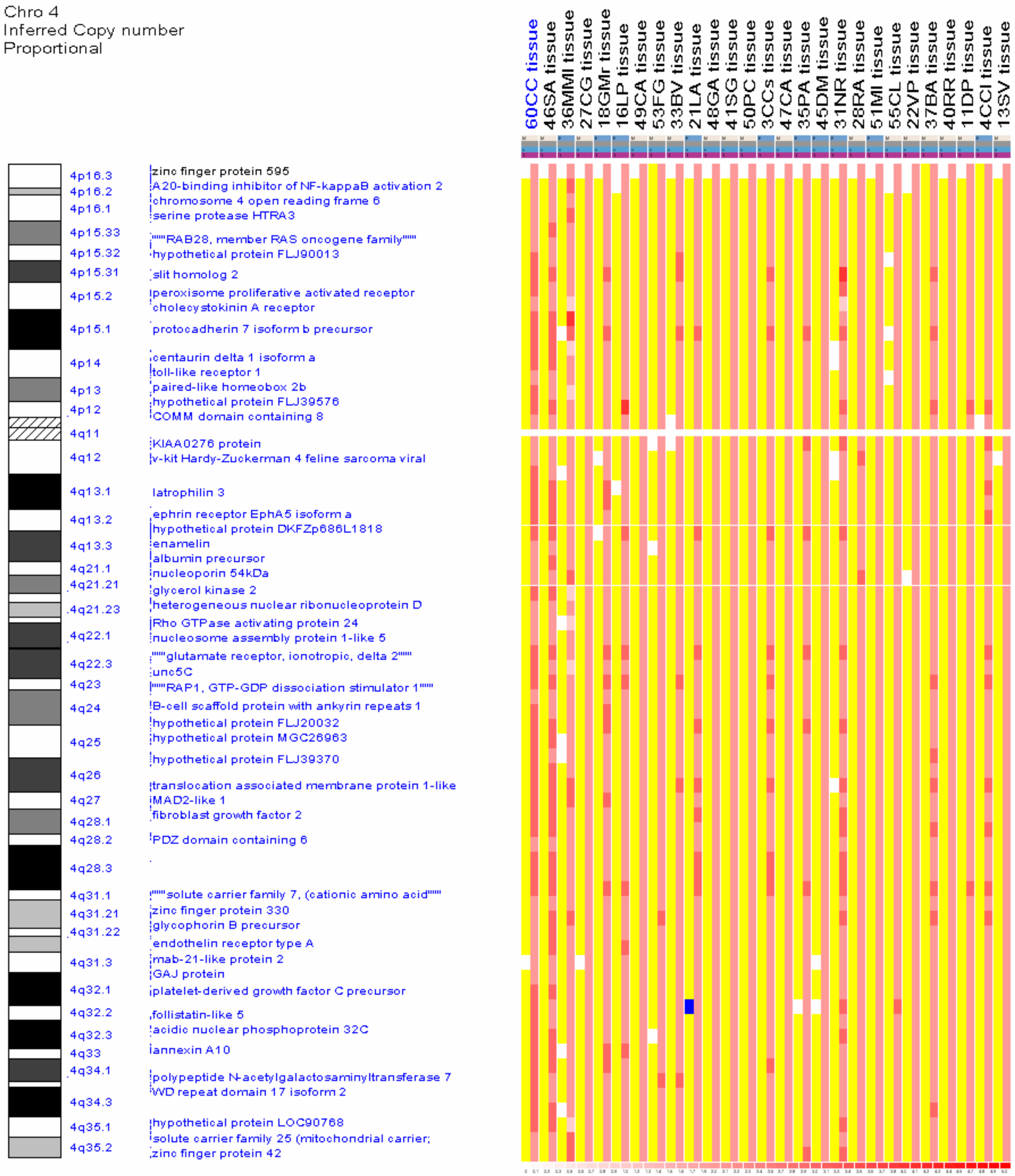
Chro 4
Inferred Copy number
Proportional
60CC tissue
46SA tissue
36MMI tissue
27CG tissue
18GMr tissue
16LP tissue
49CA tissue
53FG tissue
33BV tissue
21LA tissue
48GA tissue
41SG tissue
50PC tissue
3CCs tissue
47CA tissue
35PA tissue
45DM tissue
31NR tissue
28RA tissue
51MI tissue
55CL tissue
22VP tissue
37BA tissue
40RR tissue
11DP tissue
4CCI tissue
13SV tissue
4p16.3
4p16.2
4p16.1
4p15.33
4p15.32
4p15.31
4p15.2
4p15.1
4p14
4p13
4p12
4q11
4q12
4q13.1
4q13.2
4q13.3
4q21.1
4q21.21
4q21.23
4q22.1
4q22.3
4q23
4q24
4q25
4q26
4q27
4q28.1
4q28.2
4q28.3
4q31.1
4q31.21
4q31.22
4q31.3
4q32.1
4q32.2
4q32.3
4q33
4q34.1
4q34.3
4q35.1
4q35.2
zinc finger protein 595
A20-binding inhibitor of NF-kappaB activation 2
chromosome 4 open reading frame 6
serine protease HTRA3
""""RAB28, member RAS oncogene family""""
hypothetical protein FLJ90013
slit homolog 2
peroxisome proliferative activated receptor
cholecystokinin A receptor
protocadherin 7 isoform b precursor
centaurin delta 1 isoform a
toll-like receptor 1
paired-like homeobox 2b
hypothetical protein FLJ39576
COMM domain containing 8
KIAA0276 protein
v-kit Hardy-Zuckerman 4 feline sarcoma viral
latrophilin 3
ephrin receptor EphA5 isoform a
hypothetical protein DKFZp686L1818
enamelin
albumin precursor
nucleoporin 54kDa
glycerol kinase 2
heterogeneous nuclear ribonucleoprotein D
Rho GTPase activating protein 24
nucleosome assembly protein 1-like 5
""""glutamate receptor, ionotropic, delta 2""""
unc5C
""""RAP1, GTP-GDP dissociation stimulator 1""""
B-cell scaffold protein with ankyrin repeats 1
hypothetical protein FLJ20032
hypothetical protein MGC26963
hypothetical protein FLJ39370
translocation associated membrane protein 1-like
MAD2-like 1
fibroblast growth factor 2
PDZ domain containing 6
""""solute carrier family 7, (cationic amino acid""""
zinc finger protein 330
glycophorin B precursor
endothelin receptor type A
mab-21-like protein 2
GAJ protein
platelet-derived growth factor C precursor
follistatin-like 5
acidic nuclear phosphoprotein 32C
annexin A10
polypeptide N-acetylgalactosaminyltransferase 7
WD repeat domain 17 isoform 2
hypothetical protein LOC90768
solute carrier family 25 (mitochondrial carrier;
zinc finger protein 42

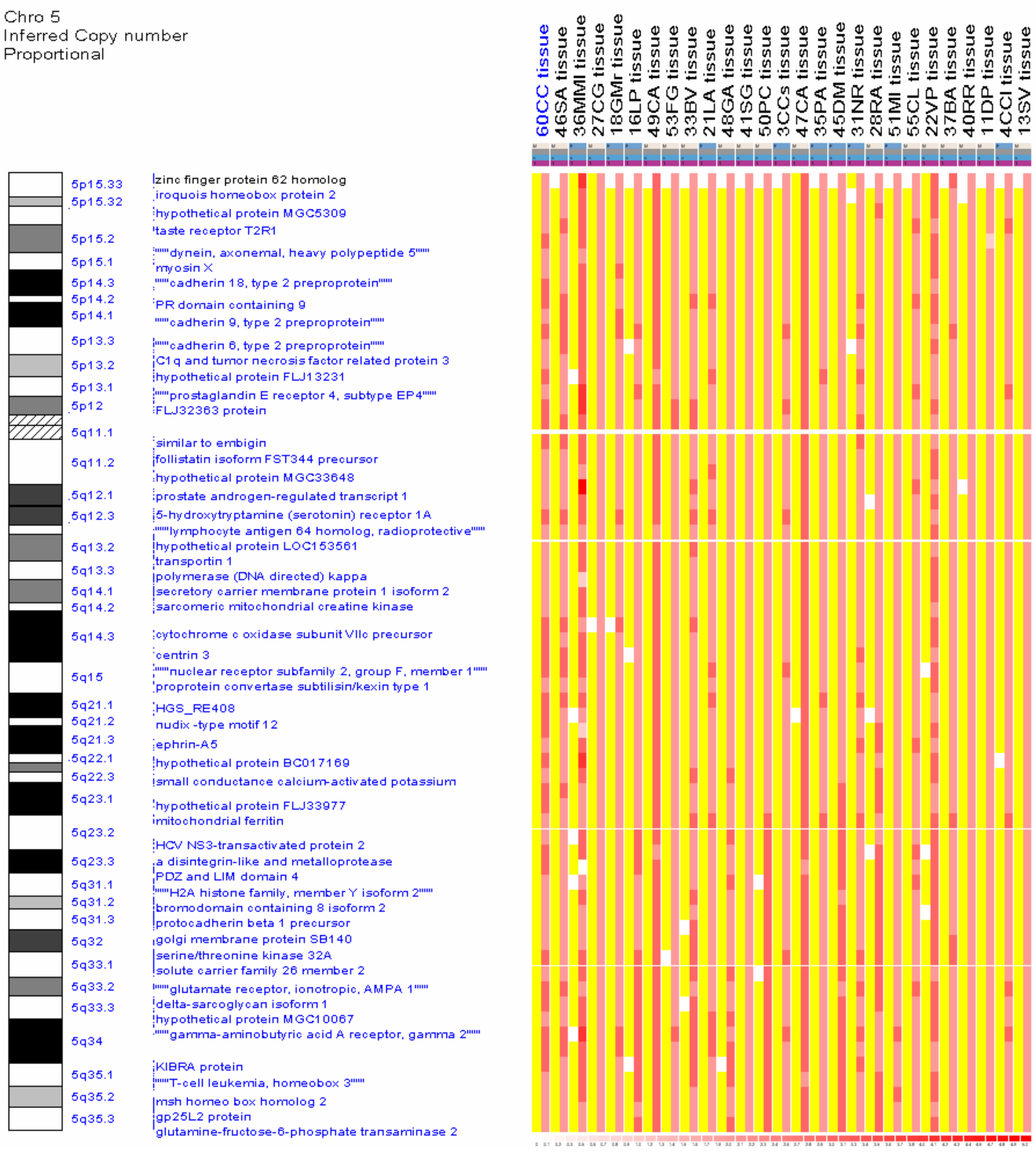

Chro 5
Inferred Copy number
Proportional
60CC tissue
46SA tissue
36MMI tissue
27CG tissue
18GMr tissue
16LP tissue
49CA tissue
53FG tissue
33BV tissue
21LA tissue
48GA tissue
41SG tissue
50PC tissue
3CCs tissue
47CA tissue
35PA tissue
45DM tissue
31NR tissue
28RA tissue
51MI tissue
55CL tissue
22VP tissue
37BA tissue
40RR tissue
11DP tissue
4CCI tissue
13SV tissue
5p15.33
5p15.32
5p15.2
5p15.1
5p14.3
5p14.2
5p14.1
5p13.3
5p13.2
5p13.1
5p12
5q11.1
5q11.2
5q12.1
5q12.3
5q13.2
5q13.3
5q14.1
5q14.2
5q14.3
5q15
5q21.1
5q21.2
5q21.3
5q22.1
5q22.3
5q23.1
5q23.2
5q23.3
5q31.1
5q31.2
5q31.3
5q32
5q33.1
5q33.2
5q33.3
5q34
5q35.1
5q35.2
5q35.3
zinc finger protein 62 homolog
iroquois homeobox protein 2
hypothetical protein MGC5309
taste receptor T2R1
"""dynein, axonemal, heavy polypeptide 5"""
myosin X
"""cadherin 18, type 2 preproprotein"""
PR domain containing 9
"""cadherin 9, type 2 preproprotein"""
"""cadherin 6, type 2 preproprotein"""
C1q and tumor necrosis factor related protein 3
hypothetical protein FLJ13231
"""prostaglandin E receptor 4, subtype EP4"""
FLJ32363 protein
similar to embigin
follistatin isoform FST344 precursor
hypothetical protein MGC33648
prostate androgen-regulated transcript 1
5-hydroxytryptamine (serotonin) receptor 1A
"""lymphocyte antigen 64 homolog, radioprotective"""
hypothetical protein LOC153561
transportin 1
polymerase (DNA directed) kappa
secretory carrier membrane protein 1 isoform 2
sarcomeric mitochondrial creatine kinase
cytochrome c oxidase subunit VIIc precursor
centrin 3
"""nuclear receptor subfamily 2, group F, member 1"""
proprotein convertase subtilisin/kexin type 1
HGS_RE408
nudix -type motif 12
ephrin-A5
hypothetical protein BC017169
small conductance calcium-activated potassium
hypothetical protein FLJ33977
mitochondrial ferritin
HCV NS3-transactivated protein 2
a disintegrin-like and metalloprotease
PDZ and LIM domain 4
"""H2A histone family, member Y isoform 2"""
bromodomain containing 8 isoform 2
protocadherin beta 1 precursor
golgi membrane protein SB140
serine/threonine kinase 32A
solute carrier family 26 member 2
"""glutamate receptor, ionotropic, AMPA 1"""
delta-sarcoglycan isoform 1
hypothetical protein MGC10067
"""gamma-aminobutyric acid A receptor, gamma 2"""
KIBRA protein
"""T-cell leukemia, homeobox 3"""
msh homeo box homolog 2
gp25L2 protein
glutamine-fructose-6-phosphate transaminase 2

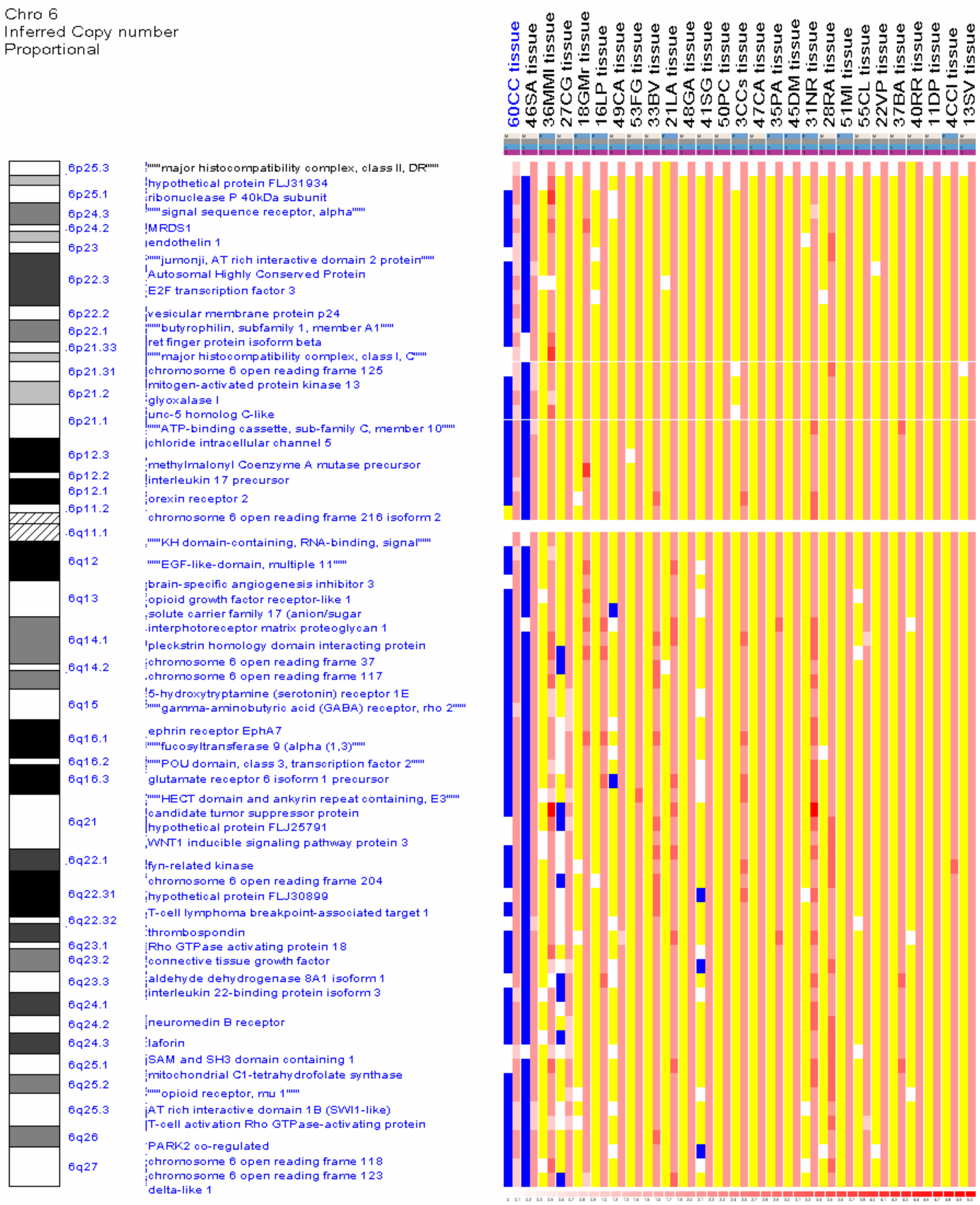

Chro 6
Inferred Copy number
Proportional
60CC tissue
46SA tissue
36MMI tissue
27CG tissue
18GMr tissue
16LP tissue
49CA tissue
53FG tissue
33BV tissue
21LA tissue
48GA tissue
41SG tissue
50PC tissue
3CCs tissue
47CA tissue
35PA tissue
45DM tissue
31NR tissue
28RA tissue
51MI tissue
55CL tissue
22VP tissue
37BA tissue
40RR tissue
11DP tissue
4CCI tissue
13SV tissue
6p25.3
6p25.1
6p24.3
6p24.2
6p23
6p22.3
6p22.2
6p22.1
6p21.33
6p21.31
6p21.2
6p21.1
6p12.3
6p12.2
6p12.1
6p11.2
6q11.1
6q12
6q13
6q14.1
6q14.2
6q15
6q16.1
6q16.2
6q16.3
6q21
6q22.1
6q22.31
6q22.32
6q23.1
6q23.2
6q23.3
6q24.1
6q24.2
6q24.3
6q25.1
6q25.2
6q25.3
6q26
6q27
"""major histocompatibility complex, class II, DR"""
hypothetical protein FLJ31934
ribonuclease P 40kDa subunit
"""signal sequence receptor, alpha"""
MRDS1
endothelin 1
"""jumonji, AT rich interactive domain 2 protein"""
Autosomal Highly Conserved Protein
E2F transcription factor 3
vesicular membrane protein p24
"""butyrophilin, subfamily 1, member A1"""
ret finger protein isoform beta
"""major histocompatibility complex, class I, C"""
chromosome 6 open reading frame 125
mitogen-activated protein kinase 13
glyoxalase I
unc-5 homolog C-like
"""ATP-binding cassette, sub-family C, member 10"""
chloride intracellular channel 5
methylmalonyl Coenzyme A mutase precursor
interleukin 17 precursor
orexin receptor 2
chromosome 6 open reading frame 216 isoform 2
"""KH domain-containing, RNA-binding, signal"""
"""EGF-like-domain, multiple 11"""
brain-specific angiogenesis inhibitor 3
opioid growth factor receptor-like 1
solute carrier family 17 (anion/sugar
interphotoreceptor matrix proteoglycan 1
pleckstrin homology domain interacting protein
chromosome 6 open reading frame 37
chromosome 6 open reading frame 117
5-hydroxytryptamine (serotonin) receptor 1E
"""gamma-aminobutyric acid (GABA) receptor, rho 2"""
ephrin receptor EphA7
"""fucosyltransferase 9 (alpha (1,3)"""
"""POU domain, class 3, transcription factor 2"""
glutamate receptor 6 isoform 1 precursor
"""HECT domain and ankyrin repeat containing, E3"""
candidate tumor suppressor protein
hypothetical protein FLJ25791
WNT1 inducible signaling pathway protein 3
fyn-related kinase
chromosome 6 open reading frame 204
hypothetical protein FLJ30899
T-cell lymphoma breakpoint-associated target 1
thrombospondin
Rho GTPase activating protein 18
connective tissue growth factor
aldehyde dehydrogenase 8A1 isoform 1
interleukin 22-binding protein isoform 3
neuromedin B receptor
laforin
SAM and SH3 domain containing 1
mitochondrial C1-tetrahydrofolate synthase
"""opioid receptor, mu 1"""
AT rich interactive domain 1B (SWI1-like)
T-cell activation Rho GTPase-activating protein
PARK2 co-regulated
chromosome 6 open reading frame 118
chromosome 6 open reading frame 123
delta-like 1

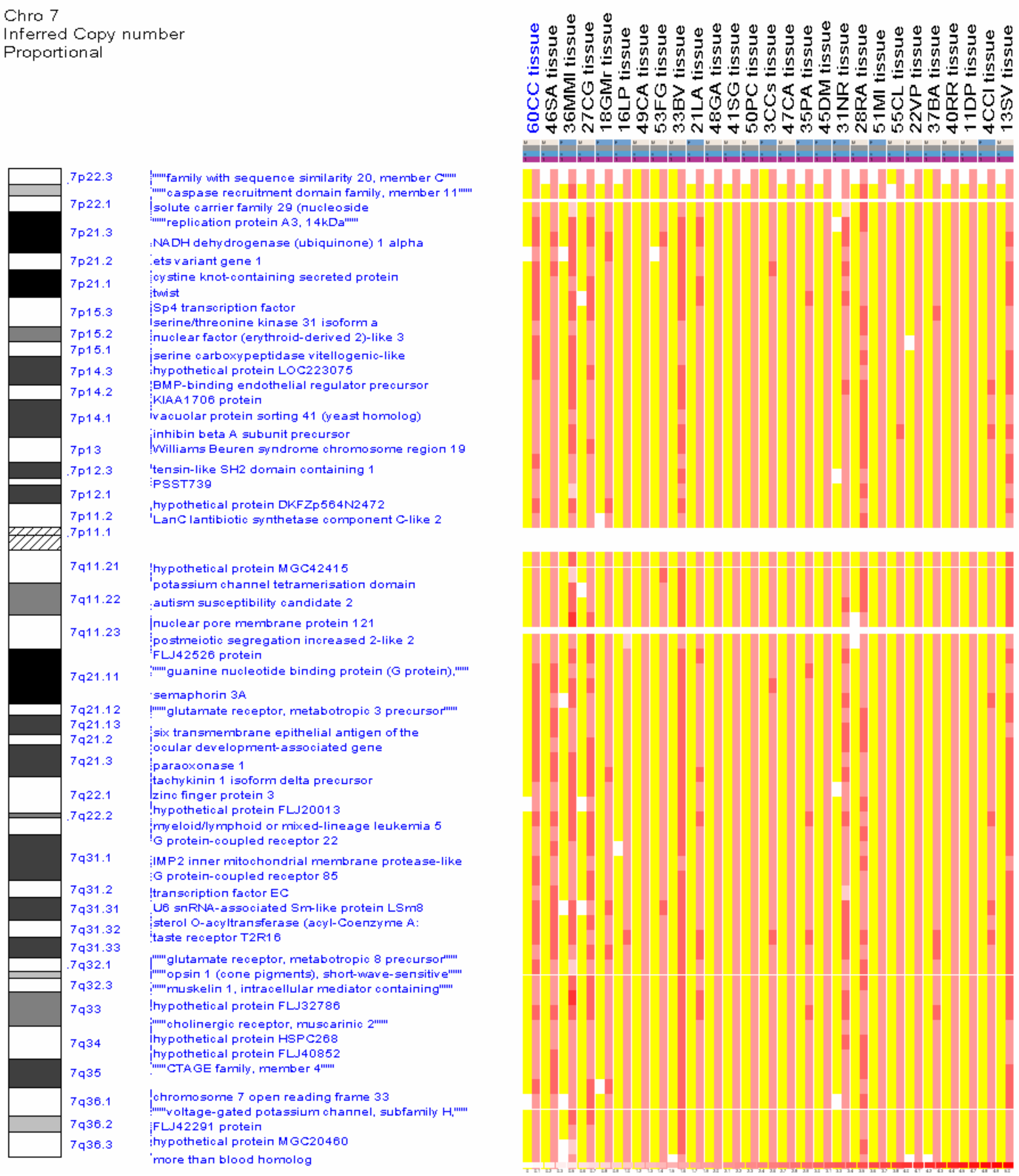

Chro 7
Inferred Copy number
Proportional
60CC tissue
46SA tissue
36MMI tissue
27CG tissue
18GMr tissue
16LP tissue
49CA tissue
53FG tissue
33BV tissue
21LA tissue
48GA tissue
41SG tissue
50PC tissue
3CCs tissue
47CA tissue
35PA tissue
45DM tissue
31NR tissue
28RA tissue
51MI tissue
55CL tissue
22VP tissue
37BA tissue
40RR tissue
11DP tissue
4CCI tissue
13SV tissue
7p22.3
7p22.1
7p21.3
7p21.2
7p21.1
7p15.3
7p15.2
7p15.1
7p14.3
7p14.2
7p14.1
7p13
7p12.3
7p12.1
7p11.2
7p11.1
7q11.21
7q11.22
7q11.23
7q21.11
7q21.12
7q21.13
7q21.2
7q21.3
7q22.1
7q22.2
7q31.1
7q31.2
7q31.31
7q31.32
7q31.33
7q32.1
7q32.3
7q33
7q34
7q35
7q36.1
7q36.2
7q36.3
"""family with sequence similarity 20, member C"""
"""caspase recruitment domain family, member 11"""
solute carrier family 29 (nucleoside
"""replication protein A3, 14kDa"""
NADH dehydrogenase (ubiquinone) 1 alpha
ets variant gene 1
cystine knot-containing secreted protein
twist
Sp4 transcription factor
serine/threonine kinase 31 isoform a
nuclear factor (erythroid-derived 2)-like 3
serine carboxypeptidase vitellogenic-like
hypothetical protein LOC223075
BMP-binding endothelial regulator precursor
KIAA1706 protein
vacuolar protein sorting 41 (yeast homolog)
inhibin beta A subunit precursor
Williams Beuren syndrome chromosome region 19
tensin-like SH2 domain containing 1
PSST739
hypothetical protein DKFZp564N2472
LanC lantibiotic synthetase component C-like 2
hypothetical protein MGC42415
potassium channel tetramerisation domain
autism susceptibility candidate 2
nuclear pore membrane protein 121
postmeiotic segregation increased 2-like 2
FLJ42526 protein
"""guanine nucleotide binding protein (G protein),"""
semaphorin 3A
"""glutamate receptor, metabotropic 3 precursor"""
six transmembrane epithelial antigen of the
ocular development-associated gene
paraoxonase 1
tachykinin 1 isoform delta precursor
zinc finger protein 3
hypothetical protein FLJ20013
myeloid/lymphoid or mixed-lineage leukemia 5
G protein-coupled receptor 22
IMP2 inner mitochondrial membrane protease-like
G protein-coupled receptor 85
transcription factor EC
U6 snRNA-associated Sm-like protein LSm8
sterol O-acyltransferase (acyl-Coenzyme A:
taste receptor T2R16
"""glutamate receptor, metabotropic 8 precursor"""
"""opsin 1 (cone pigments), short-wave-sensitive"""
"""muskelin 1, intracellular mediator containing"""
hypothetical protein FLJ32786
"""cholinergic receptor, muscarinic 2"""
hypothetical protein HSPC268
hypothetical protein FLJ40852
"""CTAGE family, member 4"""
chromosome 7 open reading frame 33
"""voltage-gated potassium channel, subfamily H,"""
FLJ42291 protein
hypothetical protein MGC20460
more than blood homolog

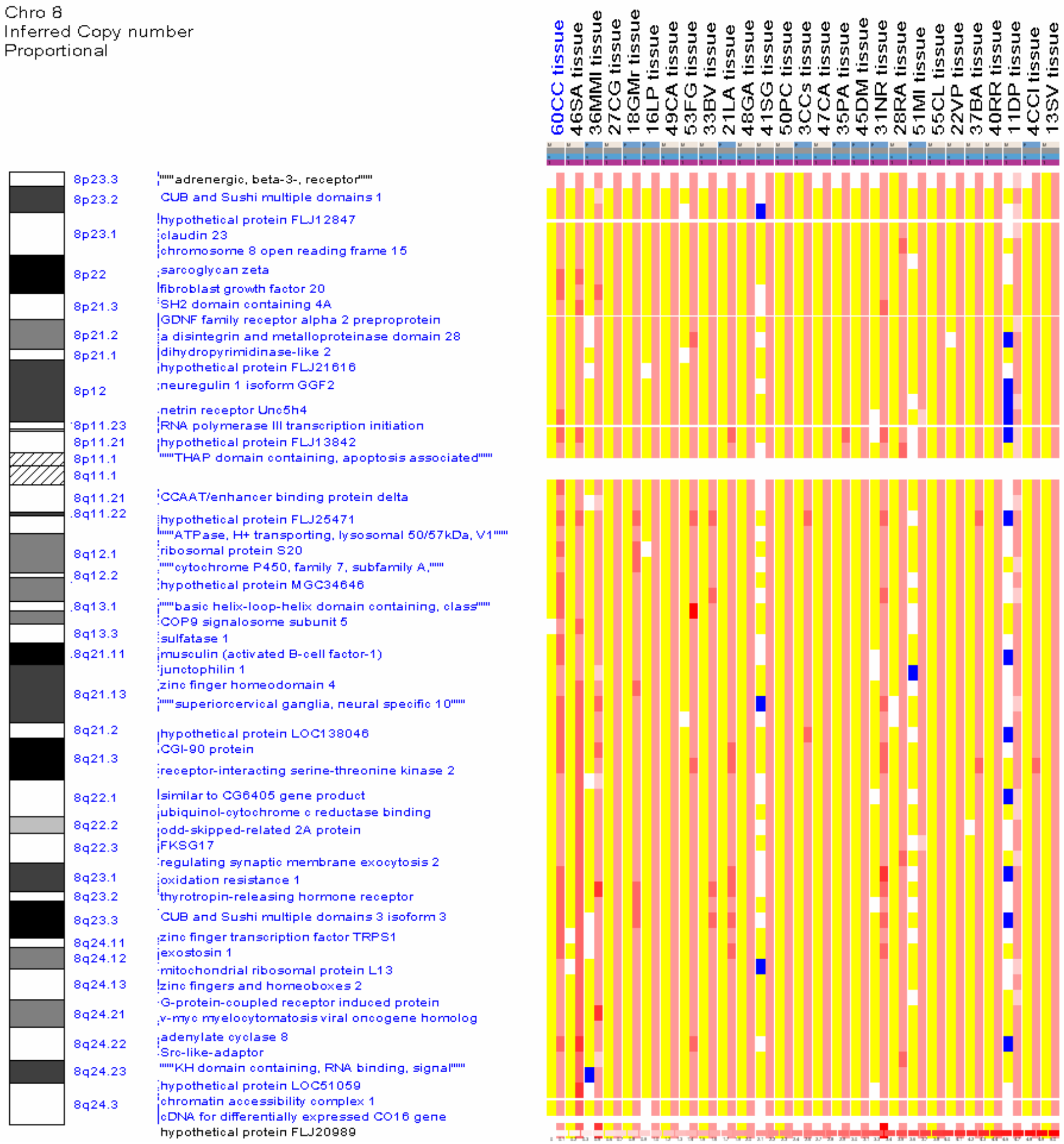

Chro 8
Inferred Copy number
Proportional
60CC tissue
46SA tissue
36MMI tissue
27CG tissue
18GMr tissue
16LP tissue
49CA tissue
53FG tissue
33BV tissue
21LA tissue
48GA tissue
41SG tissue
50PC tissue
3CCs tissue
47CA tissue
35PA tissue
45DM tissue
31NR tissue
28RA tissue
51MI tissue
55CL tissue
22VP tissue
37BA tissue
40RR tissue
11DP tissue
4CCI tissue
13SV tissue
8p23.3
8p23.2
8p23.1
8p22
8p21.3
8p21.2
8p21.1
8p12
8p11.23
8p11.21
8p11.1
8q11.1
8q11.21
8q11.22
8q12.1
8q12.2
8q13.1
8q13.3
8q21.11
8q21.13
8q21.2
8q21.3
8q22.1
8q22.2
8q22.3
8q23.1
8q23.2
8q23.3
8q24.11
8q24.12
8q24.13
8q24.21
8q24.22
8q24.23
8q24.3
"""adrenergic, beta-3-, receptor"""
CUB and Sushi multiple domains 1
hypothetical protein FLJ12847
claudin 23
chromosome 8 open reading frame 15
sarcoglycan zeta
fibroblast growth factor 20
SH2 domain containing 4A
GDNF family receptor alpha 2 preproprotein
a disintegrin and metalloproteinase domain 28
dihydropyrimidinase-like 2
hypothetical protein FLJ21616
neuregulin 1 isoform GGF2
netrin receptor Unc5h4
RNA polymerase III transcription initiation
hypothetical protein FLJ13842
"""THAP domain containing, apoptosis associated"""
CCAAT/enhancer binding protein delta
hypothetical protein FLJ25471
"""ATPase, H+ transporting, lysosomal 50/57kDa, V1"""
ribosomal protein S20
"""cytochrome P450, family 7, subfamily A,"""
hypothetical protein MGC34646
"""basic helix-loop-helix domain containing, class"""
COP9 signalosome subunit 5
sulfatase 1
musculin (activated B-cell factor-1)
junctophilin 1
zinc finger homeodomain 4
"""superiorcervical ganglia, neural specific 10"""
hypothetical protein LOC138046
CGI-90 protein
receptor-interacting serine-threonine kinase 2
similar to CG6405 gene product
ubiquinol-cytochrome c reductase binding
odd-skipped-related 2A protein
FKSG17
regulating synaptic membrane exocytosis 2
oxidation resistance 1
thyrotropin-releasing hormone receptor
CUB and Sushi multiple domains 3 isoform 3
zinc finger transcription factor TRPS1
exostosin 1
mitochondrial ribosomal protein L13
zinc fingers and homeoboxes 2
G-protein-coupled receptor induced protein
v-myc myelocytomatosis viral oncogene homolog
adenylate cyclase 8
Src-like-adaptor
"""KH domain containing, RNA binding, signal"""
hypothetical protein LOC51059
chromatin accessibility complex 1
cDNA for differentially expressed CO16 gene
hypothetical protein FLJ20989

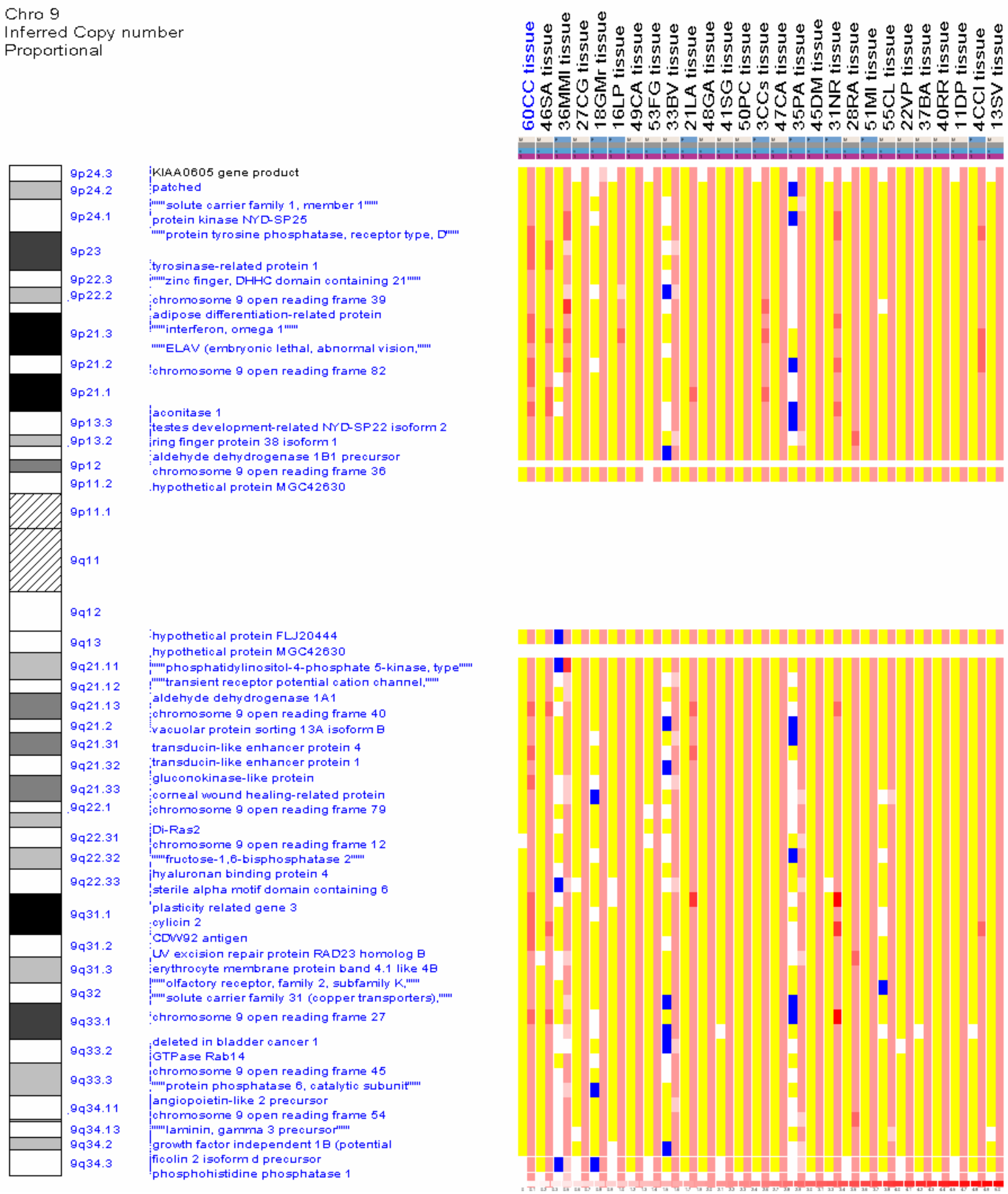
Chro 9
Inferred Copy number
Proportional
60CC tissue
46SA tissue
36MMI tissue
27CG tissue
18GMr tissue
16LP tissue
49CA tissue
53FG tissue
33BV tissue
21LA tissue
48GA tissue
41SG tissue
50PC tissue
3CCs tissue
47CA tissue
35PA tissue
45DM tissue
31NR tissue
28RA tissue
51MI tissue
55CL tissue
22VP tissue
37BA tissue
40RR tissue
11DP tissue
4CCI tissue
13SV tissue
9p24.3
9p24.2
9p24.1
9p23
9p22.3
9p22.2
9p21.3
9p21.2
9p21.1
9p13.3
9p13.2
9p12
9p11.2
9p11.1
9q11
9q12
9q13
9q21.11
9q21.12
9q21.13
9q21.2
9q21.31
9q21.32
9q21.33
9q22.1
9q22.31
9q22.32
9q22.33
9q31.1
9q31.2
9q31.3
9q32
9q33.1
9q33.2
9q33.3
9q34.11
9q34.13
9q34.2
9q34.3
KIAA0605 gene product
patched
""""solute carrier family 1, member 1""""
protein kinase NYD-SP25
""""protein tyrosine phosphatase, receptor type, D""""
tyrosinase-related protein 1
""""zinc finger, DHHC domain containing 21""""
chromosome 9 open reading frame 39
adipose differentiation-related protein
""""interferon, omega 1""""
""""ELAV (embryonic lethal, abnormal vision,""""
chromosome 9 open reading frame 82
aconitase 1
testes development-related NYD-SP22 isoform 2
ring finger protein 38 isoform 1
aldehyde dehydrogenase 1B1 precursor
chromosome 9 open reading frame 36
hypothetical protein MGC42630
hypothetical protein FLJ20444
hypothetical protein MGC42630
""""phosphatidylinositol-4-phosphate 5-kinase, type""""
""""transient receptor potential cation channel,""""
aldehyde dehydrogenase 1A1
chromosome 9 open reading frame 40
vacuolar protein sorting 13A isoform B
transducin-like enhancer protein 4
transducin-like enhancer protein 1
gluconokinase-like protein
corneal wound healing-related protein
chromosome 9 open reading frame 79
Di-Ras2
chromosome 9 open reading frame 12
""""fructose-1,6-bisphosphatase 2""""
hyaluronan binding protein 4
sterile alpha motif domain containing 6
plasticity related gene 3
cylicin 2
CDW92 antigen
UV excision repair protein RAD23 homolog B
erythrocyte membrane protein band 4.1 like 4B
""""olfactory receptor, family 2, subfamily K,""""
""""solute carrier family 31 (copper transporters),""""
chromosome 9 open reading frame 27
deleted in bladder cancer 1
GTPase Rab14
chromosome 9 open reading frame 45
""""protein phosphatase 6, catalytic subunit""""
angiopoietin-like 2 precursor
chromosome 9 open reading frame 54
""""laminin, gamma 3 precursor""""
growth factor independent 1B (potential
ficolin 2 isoform d precursor
phosphohistidine phosphatase 1

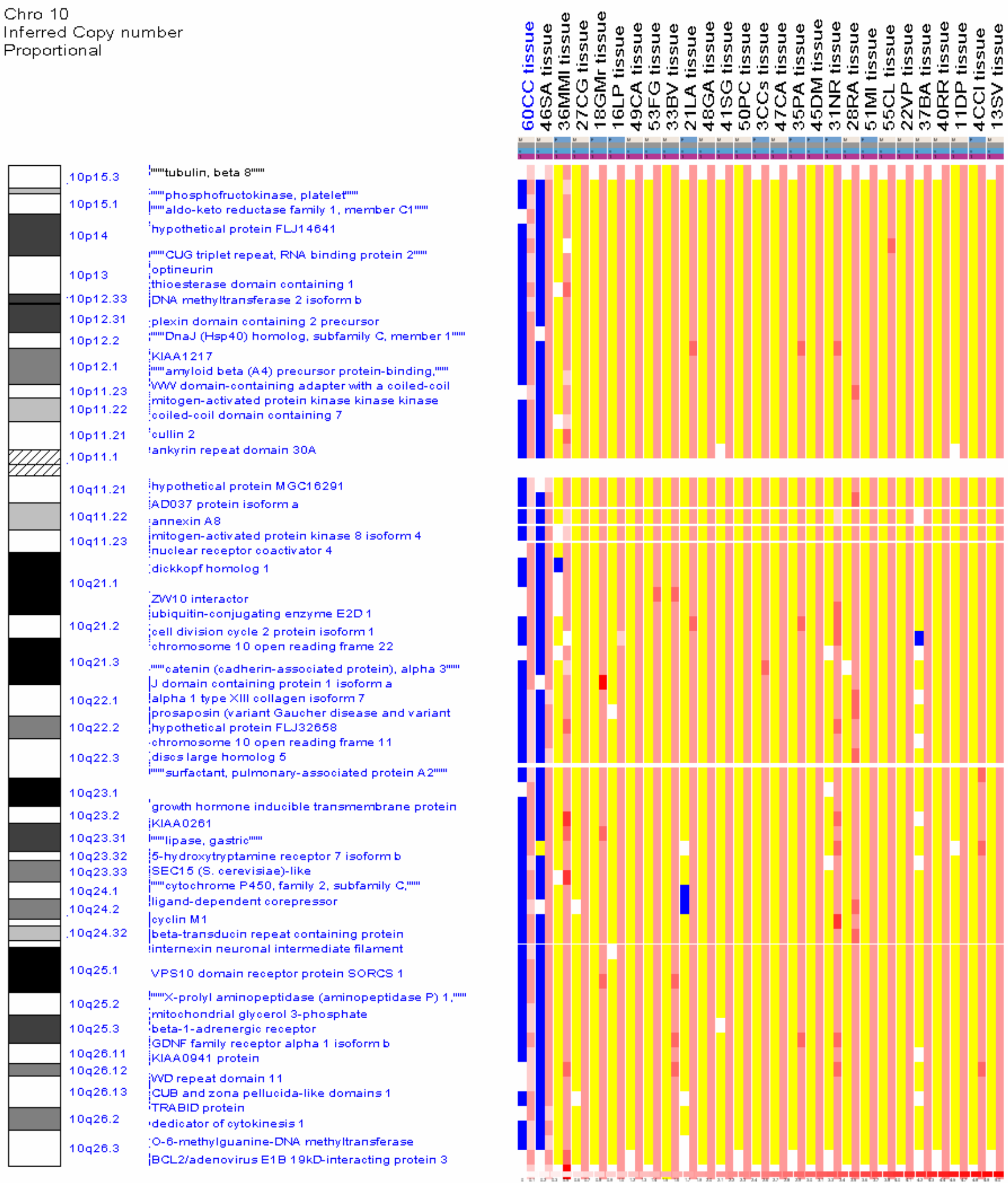
Chro 10
Inferred Copy number
Proportional
60CC tissue
46SA tissue
36MMI tissue
27CG tissue
18GMr tissue
16LP tissue
49CA tissue
53FG tissue
33BV tissue
21LA tissue
48GA tissue
41SG tissue
50PC tissue
3CCs tissue
47CA tissue
35PA tissue
45DM tissue
31NR tissue
28RA tissue
51MI tissue
55CL tissue
22VP tissue
37BA tissue
40RR tissue
11DP tissue
4CCI tissue
13SV tissue
10p15.3
10p15.1
10p14
10p13
10p12.33
10p12.31
10p12.2
10p12.1
10p11.23
10p11.22
10p11.21
10p11.1
10q11.21
10q11.22
10q11.23
10q21.1
10q21.2
10q21.3
10q22.1
10q22.2
10q22.3
10q23.1
10q23.2
10q23.31
10q23.32
10q23.33
10q24.1
10q24.2
10q24.32
10q25.1
10q25.2
10q25.3
10q26.11
10q26.12
10q26.13
10q26.2
10q26.3
"""tubulin, beta 8"""
"""phosphofructokinase, platelet"""
"""aldo-keto reductase family 1, member C1"""
hypothetical protein FLJ14641
"""CUG triplet repeat, RNA binding protein 2"""
optineurin
thioesterase domain containing 1
DNA methyltransferase 2 isoform b
plexin domain containing 2 precursor
"""DnaJ (Hsp40) homolog, subfamily C, member 1"""
KIAA1217
"""amyloid beta (A4) precursor protein-binding,"""
WW domain-containing adapter with a coiled-coil
mitogen-activated protein kinase kinase kinase
coiled-coil domain containing 7
cullin 2
ankyrin repeat domain 30A
hypothetical protein MGC16291
AD037 protein isoform a
annexin A8
mitogen-activated protein kinase 8 isoform 4
nuclear receptor coactivator 4
dickkopf homolog 1
ZW10 interactor
ubiquitin-conjugating enzyme E2D 1
cell division cycle 2 protein isoform 1
chromosome 10 open reading frame 22
"""catenin (cadherin-associated protein), alpha 3"""
J domain containing protein 1 isoform a
alpha 1 type XIII collagen isoform 7
prosaposin (variant Gaucher disease and variant
hypothetical protein FLJ32658
chromosome 10 open reading frame 11
discs large homolog 5
"""surfactant, pulmonary-associated protein A2"""
growth hormone inducible transmembrane protein
KIAA0261
"""lipase, gastric"""
5-hydroxytryptamine receptor 7 isoform b
SEC15 (S. cerevisiae)-like
"""cytochrome P450, family 2, subfamily C,"""
ligand-dependent corepressor
cyclin M1
beta-transducin repeat containing protein
internexin neuronal intermediate filament
VPS10 domain receptor protein SORCS 1
"""X-prolyl aminopeptidase (aminopeptidase P) 1,"""
mitochondrial glycerol 3-phosphate
beta-1-adrenergic receptor
GDNF family receptor alpha 1 isoform b
KIAA0941 protein
WD repeat domain 11
CUB and zona pellucida-like domains 1
TRABID protein
dedicator of cytokinesis 1
O-6-methylguanine-DNA methyltransferase
BCL2/adenovirus E1B 19kD-interacting protein 3

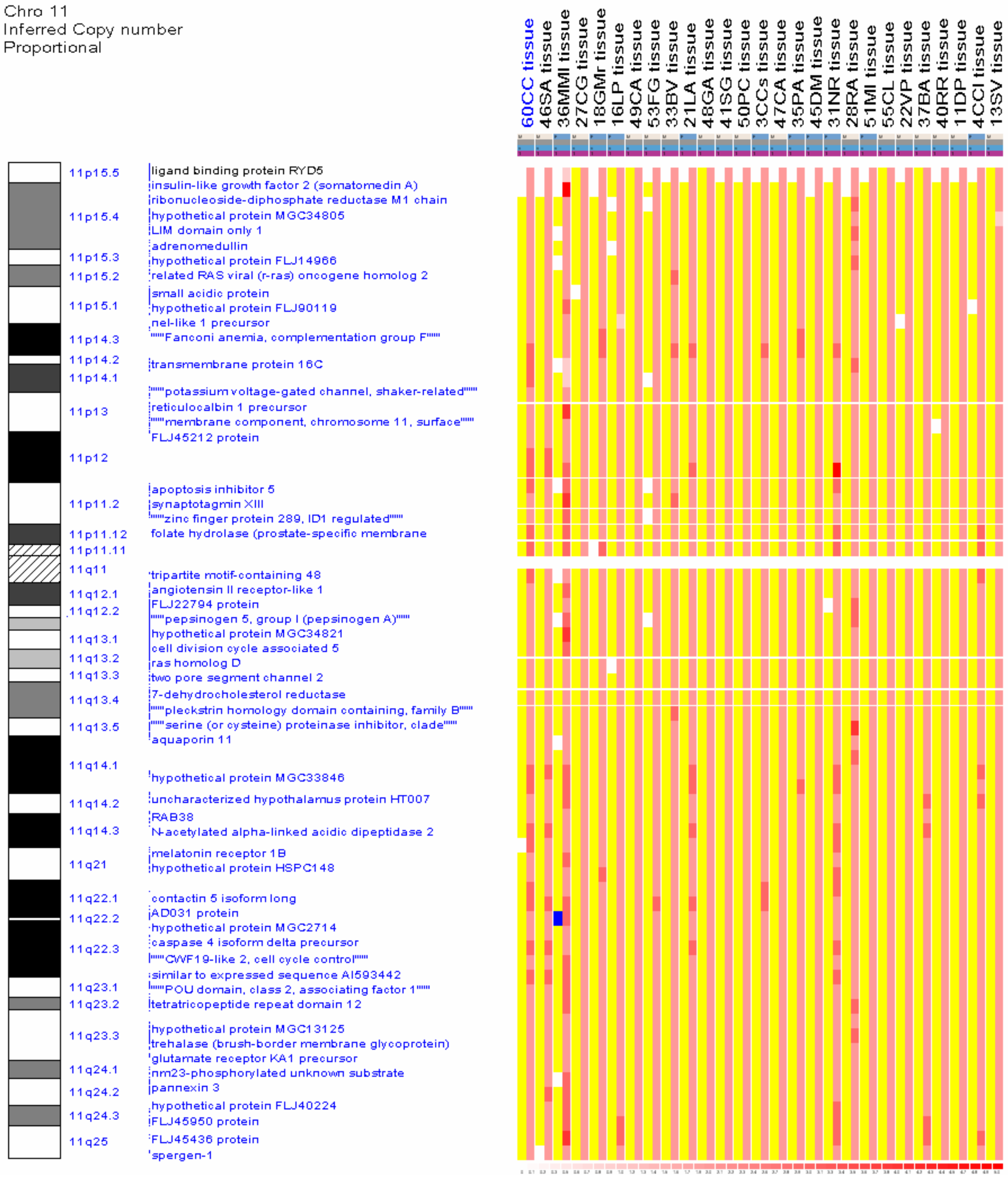
Chro 11
Inferred Copy number
Proportional
60CC tissue
46SA tissue
36MMI tissue
27CG tissue
18GMr tissue
16LP tissue
49CA tissue
53FG tissue
33BV tissue
21LA tissue
48GA tissue
41SG tissue
50PC tissue
3CCs tissue
47CA tissue
35PA tissue
45DM tissue
31NR tissue
28RA tissue
51MI tissue
55CL tissue
22VP tissue
37BA tissue
40RR tissue
11DP tissue
4CCI tissue
13SV tissue
11p15.5
11p15.4
11p15.3
11p15.2
11p15.1
11p14.3
11p14.2
11p14.1
11p13
11p12
11p11.2
11p11.12
11p11.11
11q11
11q12.1
11q12.2
11q13.1
11q13.2
11q13.3
11q13.4
11q13.5
11q14.1
11q14.2
11q14.3
11q21
11q22.1
11q22.2
11q22.3
11q23.1
11q23.2
11q23.3
11q24.1
11q24.2
11q24.3
11q25
ligand binding protein RYD5
insulin-like growth factor 2 (somatomedin A)
ribonucleoside-diphosphate reductase M1 chain
hypothetical protein MGC34805
LIM domain only 1
adrenomedullin
hypothetical protein FLJ14966
related RAS viral (r-ras) oncogene homolog 2
small acidic protein
hypothetical protein FLJ90119
nel-like 1 precursor
"""Fanconi anemia, complementation group F"""
transmembrane protein 16C
"""potassium voltage-gated channel, shaker-related"""
reticulocalbin 1 precursor
"""membrane component, chromosome 11, surface"""
FLJ45212 protein
apoptosis inhibitor 5
synaptotagmin XIII
"""zinc finger protein 289, ID1 regulated"""
folate hydrolase (prostate-specific membrane
tripartite motif-containing 48
angiotensin II receptor-like 1
FLJ22794 protein
"""pepsinogen 5, group I (pepsinogen A)"""
hypothetical protein MGC34821
cell division cycle associated 5
ras homolog D
two pore segment channel 2
7-dehydrocholesterol reductase
"""pleckstrin homology domain containing, family B"""
"""serine (or cysteine) proteinase inhibitor, clade"""
aquaporin 11
hypothetical protein MGC33846
uncharacterized hypothalamus protein HT007
RAB38
N-acetylated alpha-linked acidic dipeptidase 2
melatonin receptor 1B
hypothetical protein HSPC148
contactin 5 isoform long
AD031 protein
hypothetical protein MGC2714
caspase 4 isoform delta precursor
"""CWF19-like 2, cell cycle control"""
similar to expressed sequence AI593442
"""POU domain, class 2, associating factor 1"""
tetratricopeptide repeat domain 12
hypothetical protein MGC13125
trehalase (brush-border membrane glycoprotein)
glutamate receptor KA1 precursor
nm23-phosphorylated unknown substrate
pannexin 3
hypothetical protein FLJ40224
FLJ45950 protein
FLJ45436 protein
spergen-1

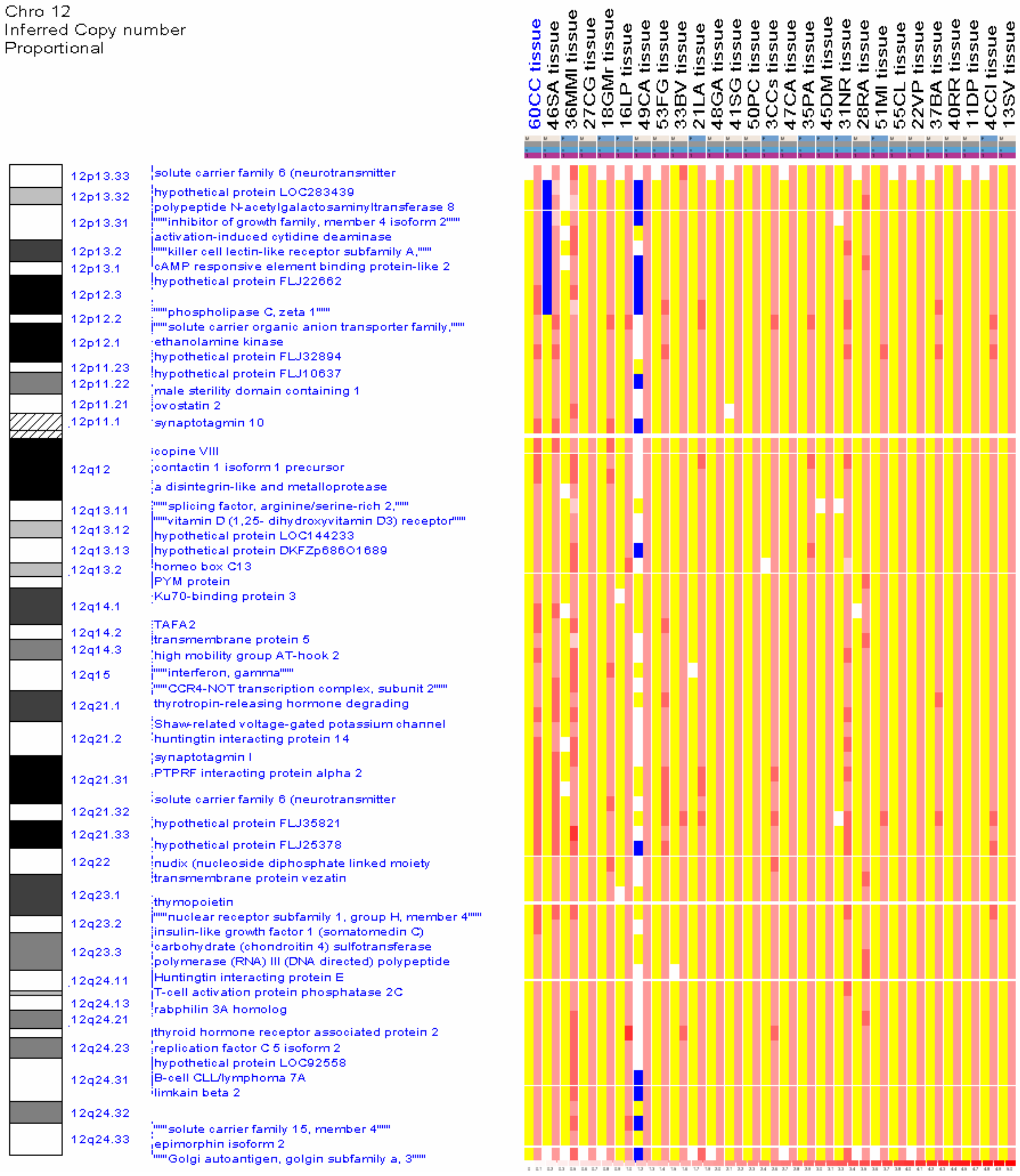
Chro 12
Inferred Copy number
Proportional
60CC tissue
46SA tissue
36MMI tissue
27CG tissue
18GMr tissue
16LP tissue
49CA tissue
53FG tissue
33BV tissue
21LA tissue
48GA tissue
41SG tissue
50PC tissue
3CCs tissue
47CA tissue
35PA tissue
45DM tissue
31NR tissue
28RA tissue
51MI tissue
55CL tissue
22VP tissue
37BA tissue
40RR tissue
11DP tissue
4CCI tissue
13SV tissue
12p13.33
12p13.32
12p13.31
12p13.2
12p13.1
12p12.3
12p12.2
12p12.1
12p11.23
12p11.22
12p11.21
12p11.1
12q12
12q13.11
12q13.12
12q13.13
12q13.2
12q14.1
12q14.2
12q14.3
12q15
12q21.1
12q21.2
12q21.31
12q21.32
12q21.33
12q22
12q23.1
12q23.2
12q23.3
12q24.11
12q24.13
12q24.21
12q24.23
12q24.31
12q24.32
12q24.33
solute carrier family 6 (neurotransmitter
hypothetical protein LOC283439
polypeptide N-acetylgalactosaminyltransferase 8
"""inhibitor of growth family, member 4 isoform 2"""
activation-induced cytidine deaminase
"""killer cell lectin-like receptor subfamily A,"""
cAMP responsive element binding protein-like 2
hypothetical protein FLJ22662
"""phospholipase C, zeta 1"""
"""solute carrier organic anion transporter family,"""
ethanolamine kinase
hypothetical protein FLJ32894
hypothetical protein FLJ10637
male sterility domain containing 1
ovostatin 2
synaptotagmin 10
copine VIII
contactin 1 isoform 1 precursor
a disintegrin-like and metalloprotease
"""splicing factor, arginine/serine-rich 2,"""
"""vitamin D (1,25- dihydroxyvitamin D3) receptor"""
hypothetical protein LOC144233
hypothetical protein DKFZp686O1689
homeo box C13
PYM protein
Ku70-binding protein 3
TAFA2
transmembrane protein 5
high mobility group AT-hook 2
"""interferon, gamma"""
"""CCR4-NOT transcription complex, subunit 2"""
thyrotropin-releasing hormone degrading
Shaw-related voltage-gated potassium channel
huntingtin interacting protein 14
synaptotagmin I
PTPRF interacting protein alpha 2
solute carrier family 6 (neurotransmitter
hypothetical protein FLJ35821
hypothetical protein FLJ25378
nudix (nucleoside diphosphate linked moiety
transmembrane protein vezatin
thymopoietin
"""nuclear receptor subfamily 1, group H, member 4"""
insulin-like growth factor 1 (somatomedin C)
carbohydrate (chondroitin 4) sulfotransferase
polymerase (RNA) III (DNA directed) polypeptide
Huntingtin interacting protein E
T-cell activation protein phosphatase 2C
rabphilin 3A homolog
thyroid hormone receptor associated protein 2
replication factor C 5 isoform 2
hypothetical protein LOC92558
B-cell CLL/lymphoma 7A
limkain beta 2
"""solute carrier family 15, member 4"""
epimorphin isoform 2
"""Golgi autoantigen, golgin subfamily a, 3"""

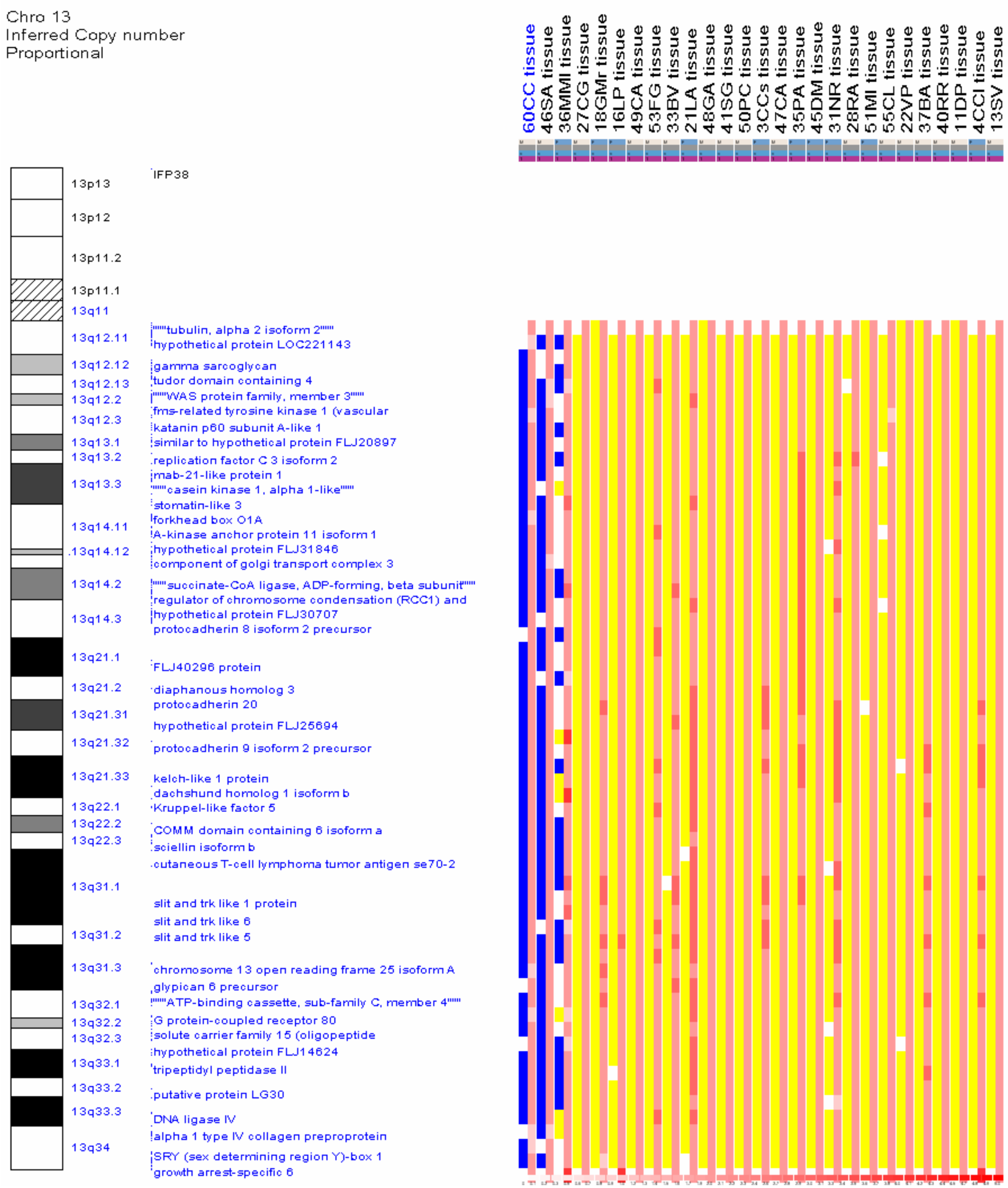

Chro 13
Inferred Copy number
Proportional
60CC tissue
46SA tissue
36MMI tissue
27CG tissue
18GMr tissue
16LP tissue
49CA tissue
53FG tissue
33BV tissue
21LA tissue
48GA tissue
41SG tissue
50PC tissue
3CCs tissue
47CA tissue
35PA tissue
45DM tissue
31NR tissue
28RA tissue
51MI tissue
55CL tissue
22VP tissue
37BA tissue
40RR tissue
11DP tissue
4CCI tissue
13SV tissue
13p13
13p12
13p11.2
13p11.1
13q11
13q12.11
13q12.12
13q12.13
13q12.2
13q12.3
13q13.1
13q13.2
13q13.3
13q14.11
13q14.12
13q14.2
13q14.3
13q21.1
13q21.2
13q21.31
13q21.32
13q21.33
13q22.1
13q22.2
13q22.3
13q31.1
13q31.2
13q31.3
13q32.1
13q32.2
13q32.3
13q33.1
13q33.2
13q33.3
13q34
IFP38
"""tubulin, alpha 2 isoform 2"""
hypothetical protein LOC221143
gamma sarcoglycan
tudor domain containing 4
"""WAS protein family, member 3"""
fms-related tyrosine kinase 1 (vascular
katanin p60 subunit A-like 1
similar to hypothetical protein FLJ20897
replication factor C 3 isoform 2
mab-21-like protein 1
"""casein kinase 1, alpha 1-like"""
stomatin-like 3
forkhead box O1A
A-kinase anchor protein 11 isoform 1
hypothetical protein FLJ31846
component of golgi transport complex 3
"""succinate-CoA ligase, ADP-forming, beta subunit"""
regulator of chromosome condensation (RCC1) and
hypothetical protein FLJ30707
protocadherin 8 isoform 2 precursor
FLJ40296 protein
diaphanous homolog 3
protocadherin 20
hypothetical protein FLJ25694
protocadherin 9 isoform 2 precursor
kelch-like 1 protein
dachshund homolog 1 isoform b
Kruppel-like factor 5
COMM domain containing 6 isoform a
sciellin isoform b
cutaneous T-cell lymphoma tumor antigen se70-2
slit and trk like 1 protein
slit and trk like 6
slit and trk like 5
chromosome 13 open reading frame 25 isoform A
glypican 6 precursor
"""ATP-binding cassette, sub-family C, member 4"""
G protein-coupled receptor 80
solute carrier family 15 (oligopeptide
hypothetical protein FLJ14624
tripeptidyl peptidase II
putative protein LG30
DNA ligase IV
alpha 1 type IV collagen preproprotein
SRY (sex determining region Y)-box 1
growth arrest-specific 6

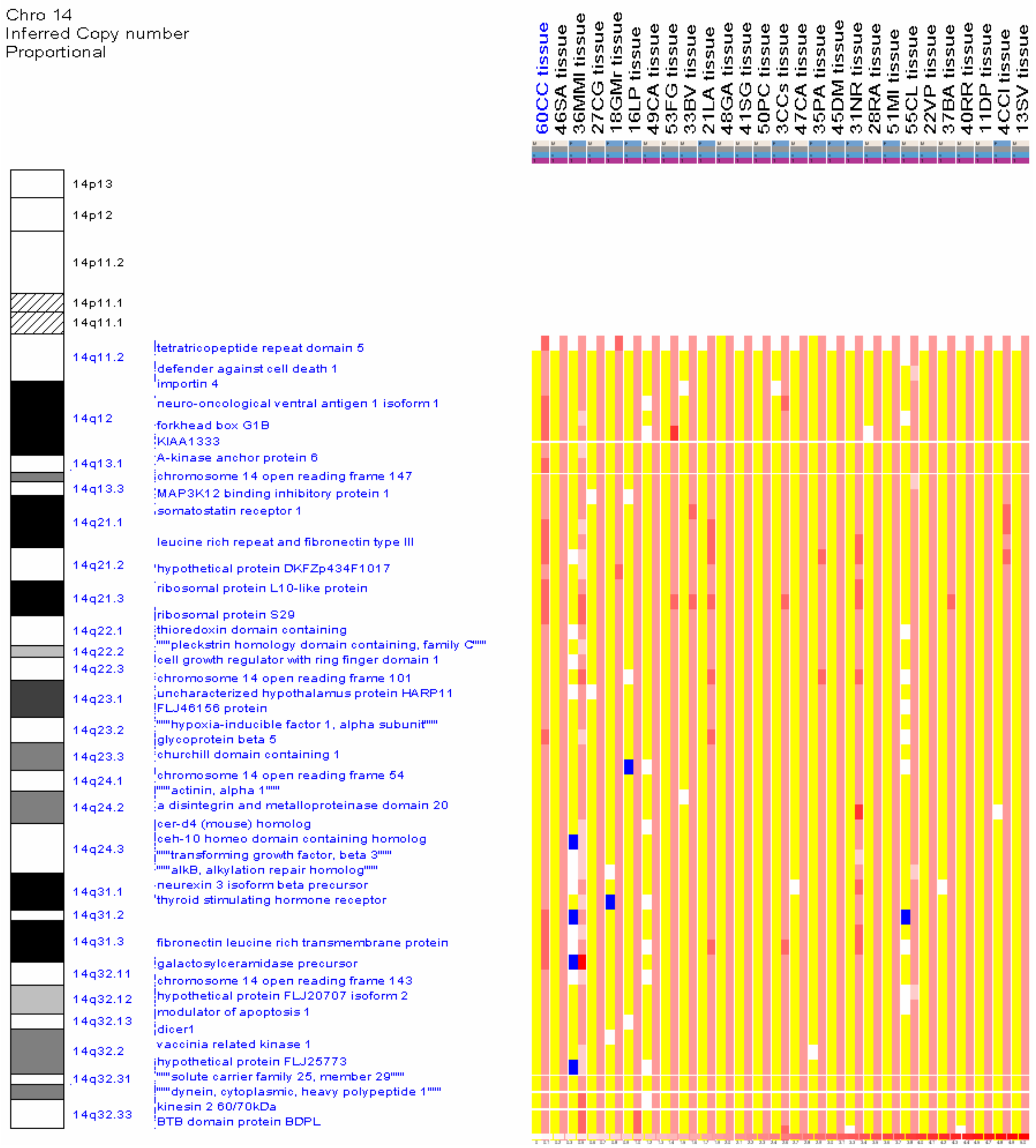

Chro 14
Inferred Copy number
Proportional
60CC tissue
46SA tissue
36MMI tissue
27CG tissue
18GMr tissue
16LP tissue
49CA tissue
53FG tissue
33BV tissue
21LA tissue
48GA tissue
41SG tissue
50PC tissue
3CCs tissue
47CA tissue
35PA tissue
45DM tissue
31NR tissue
28RA tissue
51MI tissue
55CL tissue
22VP tissue
37BA tissue
40RR tissue
11DP tissue
4CCI tissue
13SV tissue
14p13
14p12
14p11.2
14p11.1
14q11.1
14q11.2
14q12
14q13.1
14q13.3
14q21.1
14q21.2
14q21.3
14q22.1
14q22.2
14q22.3
14q23.1
14q23.2
14q23.3
14q24.1
14q24.2
14q24.3
14q31.1
14q31.2
14q31.3
14q32.11
14q32.12
14q32.13
14q32.2
14q32.31
14q32.33
tetratricopeptide repeat domain 5
defender against cell death 1
importin 4
neuro-oncological ventral antigen 1 isoform 1
forkhead box G1B
KIAA1333
A-kinase anchor protein 6
chromosome 14 open reading frame 147
MAP3K12 binding inhibitory protein 1
somatostatin receptor 1
leucine rich repeat and fibronectin type III
hypothetical protein DKFZp434F1017
ribosomal protein L10-like protein
ribosomal protein S29
thioredoxin domain containing
"""pleckstrin homology domain containing, family C"""
cell growth regulator with ring finger domain 1
chromosome 14 open reading frame 101
uncharacterized hypothalamus protein HARP11
FLJ46156 protein
"""hypoxia-inducible factor 1, alpha subunit"""
glycoprotein beta 5
churchill domain containing 1
chromosome 14 open reading frame 54
"""actinin, alpha 1"""
a disintegrin and metalloproteinase domain 20
cer-d4 (mouse) homolog
ceh-10 homeo domain containing homolog
"""transforming growth factor, beta 3"""
"""alkB, alkylation repair homolog"""
neurexin 3 isoform beta precursor
thyroid stimulating hormone receptor
fibronectin leucine rich transmembrane protein
galactosylceramidase precursor
chromosome 14 open reading frame 143
hypothetical protein FLJ20707 isoform 2
modulator of apoptosis 1
dicer1
vaccinia related kinase 1
hypothetical protein FLJ25773
"""solute carrier family 25, member 29"""
"""dynein, cytoplasmic, heavy polypeptide 1"""
kinesin 2 60/70kDa
BTB domain protein BDPL

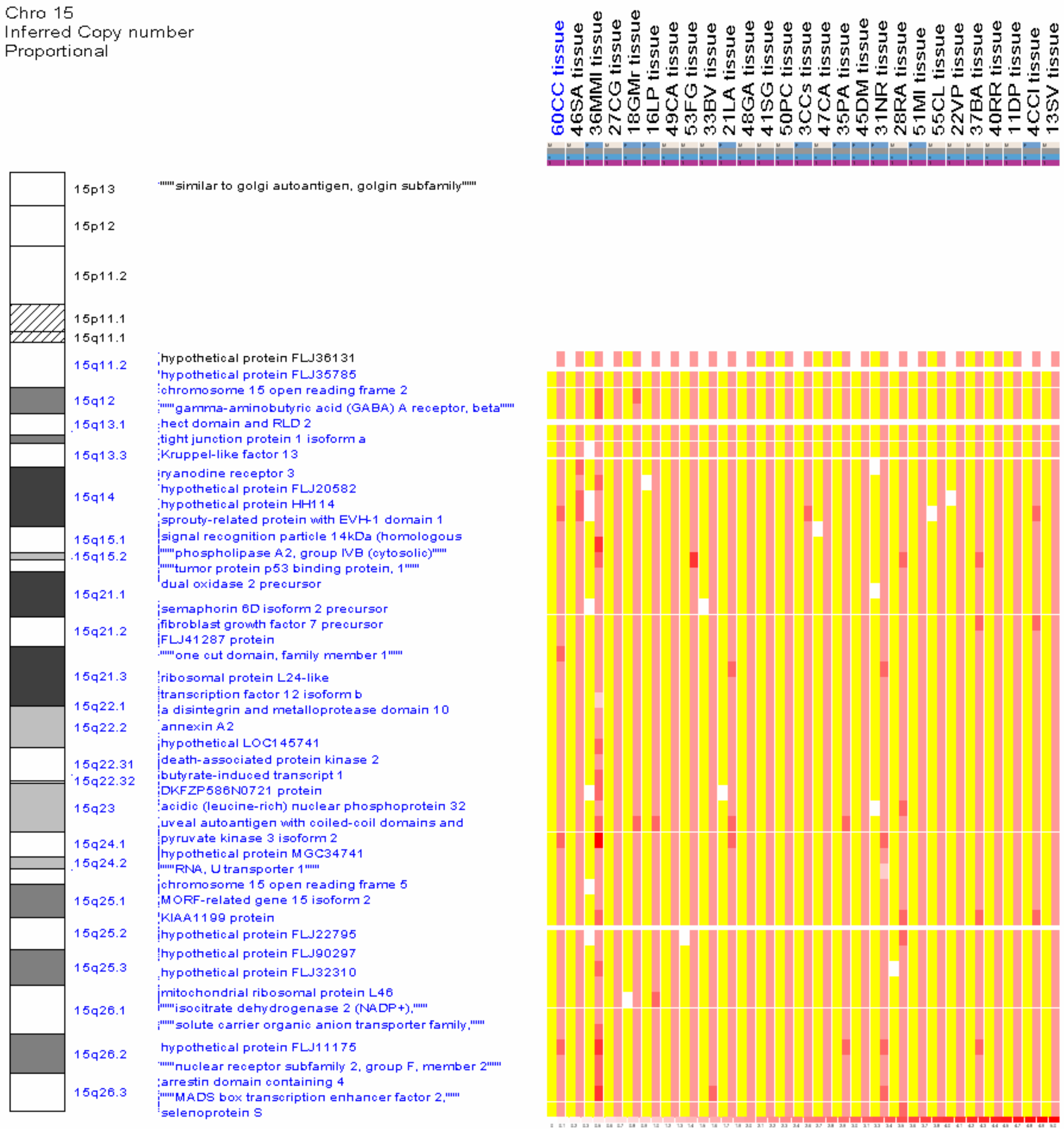
Chro 15
Inferred Copy number
Proportional
60CC tissue
46SA tissue
36MMI tissue
27CG tissue
18GMr tissue
16LP tissue
49CA tissue
53FG tissue
33BV tissue
21LA tissue
48GA tissue
41SG tissue
50PC tissue
3CCs tissue
47CA tissue
35PA tissue
45DM tissue
31NR tissue
28RA tissue
51MI tissue
55CL tissue
22VP tissue
37BA tissue
40RR tissue
11DP tissue
4CCI tissue
13SV tissue
15p13
15p12
15p11.2
15p11.1
15q11.1
15q11.2
15q12
15q13.1
15q13.3
15q14
15q15.1
15q15.2
15q21.1
15q21.2
15q21.3
15q22.1
15q22.2
15q22.31
15q22.32
15q23
15q24.1
15q24.2
15q25.1
15q25.2
15q25.3
15q26.1
15q26.2
15q26.3
""""similar to golgi autoantigen, golgin subfamily""""
hypothetical protein FLJ36131
hypothetical protein FLJ35785
chromosome 15 open reading frame 2
""""gamma-aminobutyric acid (GABA) A receptor, beta""""
hect domain and RLD 2
tight junction protein 1 isoform a
Kruppel-like factor 13
ryanodine receptor 3
hypothetical protein FLJ20582
hypothetical protein HH114
sprouty-related protein with EVH-1 domain 1
signal recognition particle 14kDa (homologous
""""phospholipase A2, group IVB (cytosolic)""""
""""tumor protein p53 binding protein, 1""""
dual oxidase 2 precursor
semaphorin 6D isoform 2 precursor
fibroblast growth factor 7 precursor
FLJ41287 protein
""""one cut domain, family member 1""""
ribosomal protein L24-like
transcription factor 12 isoform b
a disintegrin and metalloprotease domain 10
annexin A2
hypothetical LOC145741
death-associated protein kinase 2
butyrate-induced transcript 1
DKFZP586N0721 protein
acidic (leucine-rich) nuclear phosphoprotein 32
uveal autoantigen with coiled-coil domains and
pyruvate kinase 3 isoform 2
hypothetical protein MGC34741
""""RNA, U transporter 1""""
chromosome 15 open reading frame 5
MORF-related gene 15 isoform 2
KIAA1199 protein
hypothetical protein FLJ22795
hypothetical protein FLJ90297
hypothetical protein FLJ32310
mitochondrial ribosomal protein L46
""""isocitrate dehydrogenase 2 (NADP+),""""
""""solute carrier organic anion transporter family,""""
hypothetical protein FLJ11175
""""nuclear receptor subfamily 2, group F, member 2""""
arrestin domain containing 4
""""MADS box transcription enhancer factor 2,""""
selenoprotein S

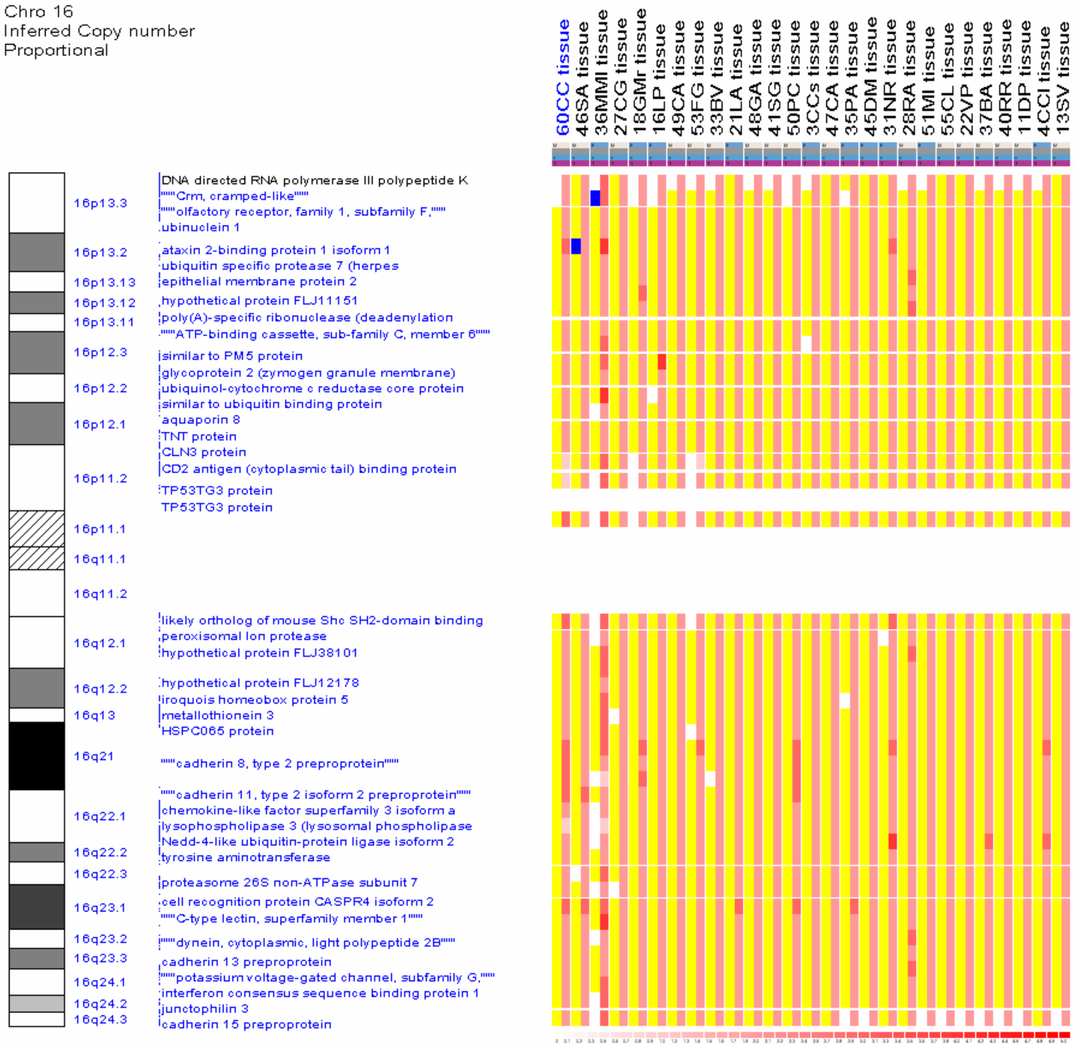
Chro 16
Inferred Copy number
Proportional
60CC tissue
46SA tissue
36MMI tissue
27CG tissue
18GMr tissue
16LP tissue
49CA tissue
53FG tissue
33BV tissue
21LA tissue
48GA tissue
41SG tissue
50PC tissue
3CCs tissue
47CA tissue
35PA tissue
45DM tissue
31NR tissue
28RA tissue
51MI tissue
55CL tissue
22VP tissue
37BA tissue
40RR tissue
11DP tissue
4CCI tissue
13SV tissue
16p13.3
16p13.2
16p13.13
16p13.12
16p13.11
16p12.3
16p12.2
16p12.1
16p11.2
16p11.1
16q11.1
16q11.2
16q12.1
16q12.2
16q13
16q21
16q22.1
16q22.2
16q22.3
16q23.1
16q23.2
16q23.3
16q24.1
16q24.2
16q24.3
DNA directed RNA polymerase III polypeptide K
""""Crm, cramped-like""""
""""olfactory receptor, family 1, subfamily F,""""
ubinuclein 1
ataxin 2-binding protein 1 isoform 1
ubiquitin specific protease 7 (herpes
epithelial membrane protein 2
hypothetical protein FLJ11151
poly(A)-specific ribonuclease (deadenylation
""""ATP-binding cassette, sub-family C, member 6""""
similar to PM5 protein
glycoprotein 2 (zymogen granule membrane)
ubiquinol-cytochrome c reductase core protein
similar to ubiquitin binding protein
aquaporin 8
TNT protein
CLN3 protein
CD2 antigen (cytoplasmic tail) binding protein
TP53TG3 protein
TP53TG3 protein
likely ortholog of mouse Shc SH2-domain binding
peroxisomal lon protease
hypothetical protein FLJ38101
hypothetical protein FLJ12178
iroquois homeobox protein 5
metallothionein 3
HSPC065 protein
""""cadherin 8, type 2 preproprotein""""
""""cadherin 11, type 2 isoform 2 preproprotein""""
chemokine-like factor superfamily 3 isoform a
lysophospholipase 3 (lysosomal phospholipase
Nedd-4-like ubiquitin-protein ligase isoform 2
tyrosine aminotransferase
proteasome 26S non-ATPase subunit 7
cell recognition protein CASPR4 isoform 2
""""C-type lectin, superfamily member 1""""
""""dynein, cytoplasmic, light polypeptide 2B""""
cadherin 13 preproprotein
""""potassium voltage-gated channel, subfamily G,""""
interferon consensus sequence binding protein 1
junctophilin 3
cadherin 15 preproprotein

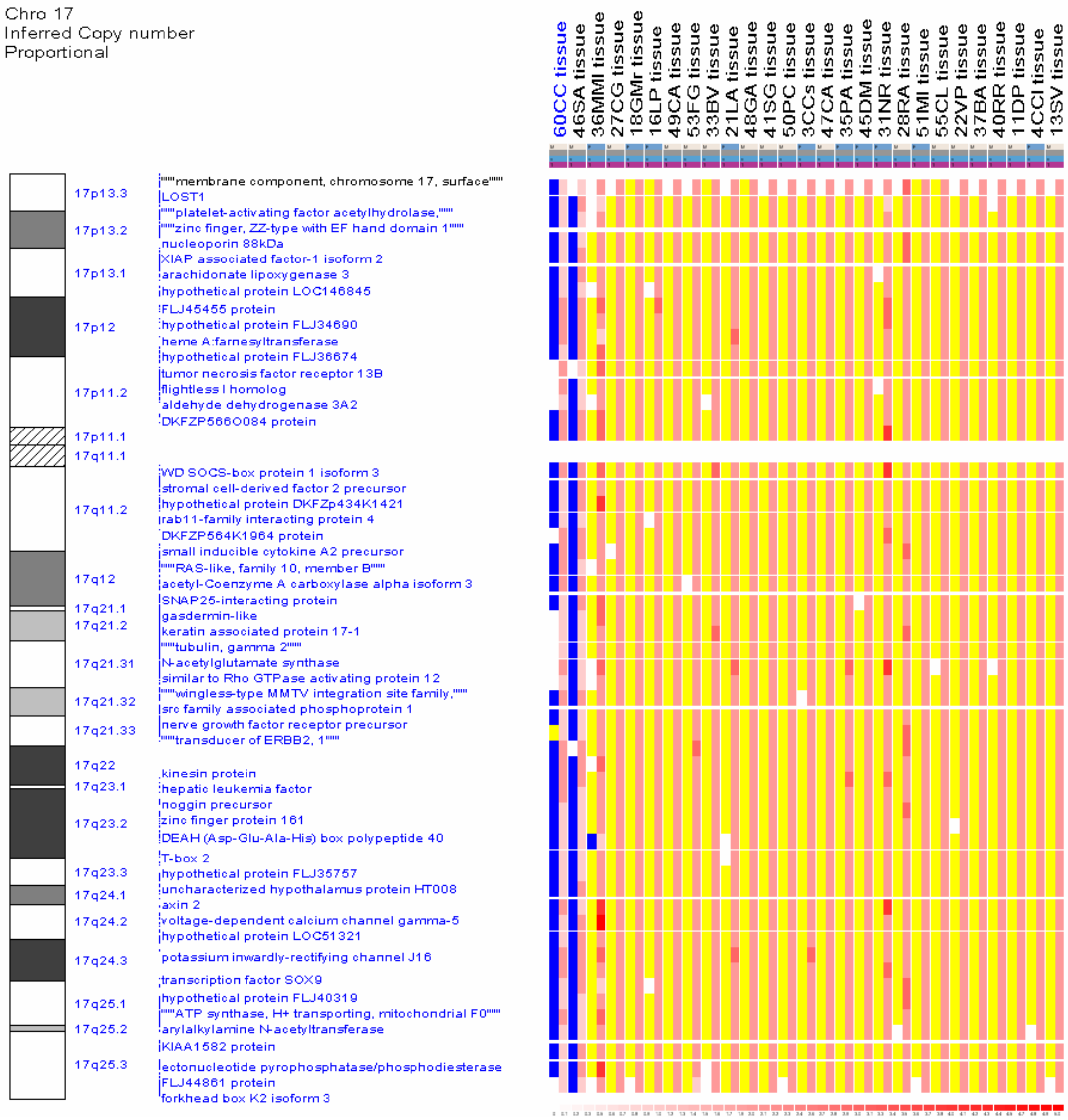

Chro 17
Inferred Copy number
Proportional
60CC tissue
46SA tissue
36MMI tissue
27CG tissue
18GMr tissue
16LP tissue
49CA tissue
53FG tissue
33BV tissue
21LA tissue
48GA tissue
41SG tissue
50PC tissue
3CCs tissue
47CA tissue
35PA tissue
45DM tissue
31NR tissue
28RA tissue
51MI tissue
55CL tissue
22VP tissue
37BA tissue
40RR tissue
11DP tissue
4CCI tissue
13SV tissue
17p13.3
17p13.2
17p13.1
17p12
17p11.2
17p11.1
17q11.1
17q11.2
17q12
17q21.1
17q21.2
17q21.31
17q21.32
17q21.33
17q22
17q23.1
17q23.2
17q23.3
17q24.1
17q24.2
17q24.3
17q25.1
17q25.2
17q25.3
""""membrane component, chromosome 17, surface""""
LOST1
""""platelet-activating factor acetylhydrolase,""""
""""zinc finger, ZZ-type with EF hand domain 1""""
nucleoporin 88kDa
XIAP associated factor-1 isoform 2
arachidonate lipoxygenase 3
hypothetical protein LOC146845
FLJ45455 protein
hypothetical protein FLJ34690
heme A:farnesyltransferase
hypothetical protein FLJ36674
tumor necrosis factor receptor 13B
flightless I homolog
aldehyde dehydrogenase 3A2
DKFZP566O084 protein
WD SOCS-box protein 1 isoform 3
stromal cell-derived factor 2 precursor
hypothetical protein DKFZp434K1421
rab11-family interacting protein 4
DKFZP564K1964 protein
small inducible cytokine A2 precursor
""""RAS-like, family 10, member B""""
acetyl-Coenzyme A carboxylase alpha isoform 3
SNAP25-interacting protein
gasdermin-like
keratin associated protein 17-1
""""tubulin, gamma 2""""
N-acetylglutamate synthase
similar to Rho GTPase activating protein 12
""""wingless-type MMTV integration site family,""""
src family associated phosphoprotein 1
nerve growth factor receptor precursor
""""transducer of ERBB2, 1""""
kinesin protein
hepatic leukemia factor
noggin precursor
zinc finger protein 161
DEAH (Asp-Glu-Ala-His) box polypeptide 40
T-box 2
hypothetical protein FLJ35757
uncharacterized hypothalamus protein HT008
axin 2
voltage-dependent calcium channel gamma-5
hypothetical protein LOC51321
potassium inwardly-rectifying channel J16
transcription factor SOX9
hypothetical protein FLJ40319
""""ATP synthase, H+ transporting, mitochondrial F0""""
arylalkylamine N-acetyltransferase
KIAA1582 protein
ectonucleotide pyrophosphatase/phosphodiesterase
FLJ44861 protein
forkhead box K2 isoform 3

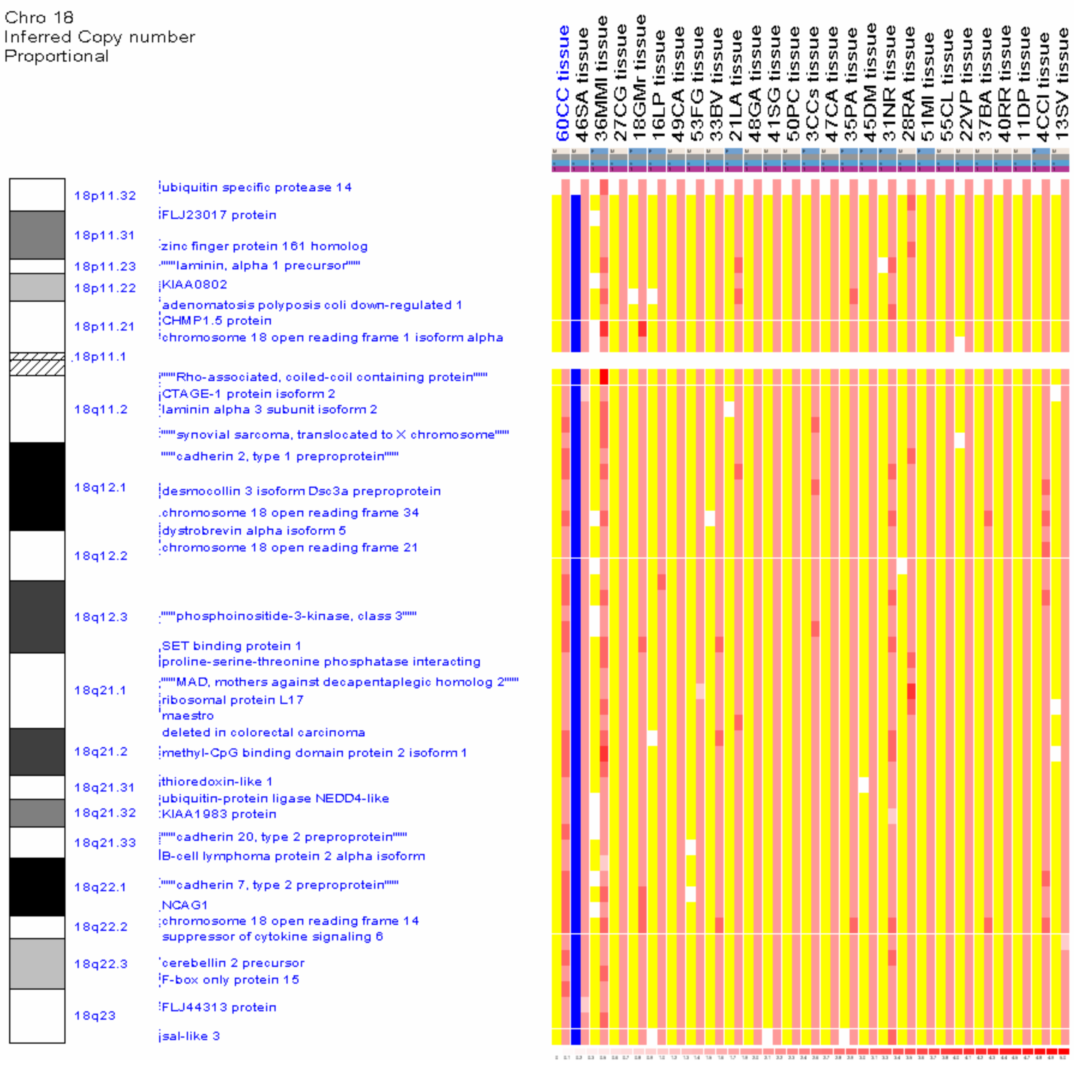

Chro 18
Inferred Copy number
Proportional
60CC tissue
46SA tissue
36MMI tissue
27CG tissue
18GMr tissue
16LP tissue
49CA tissue
53FG tissue
33BV tissue
21LA tissue
48GA tissue
41SG tissue
50PC tissue
3CCs tissue
47CA tissue
35PA tissue
45DM tissue
31NR tissue
28RA tissue
51MI tissue
55CL tissue
22VP tissue
37BA tissue
40RR tissue
11DP tissue
4CCI tissue
13SV tissue
18p11.32
18p11.31
18p11.23
18p11.22
18p11.21
18p11.1
18q11.2
18q12.1
18q12.2
18q12.3
18q21.1
18q21.2
18q21.31
18q21.32
18q21.33
18q22.1
18q22.2
18q22.3
18q23
ubiquitin specific protease 14
FLJ23017 protein
zinc finger protein 161 homolog
"""laminin, alpha 1 precursor"""
KIAA0802
adenomatosis polyposis coli down-regulated 1
CHMP1.5 protein
chromosome 18 open reading frame 1 isoform alpha
"""Rho-associated, coiled-coil containing protein"""
CTAGE-1 protein isoform 2
laminin alpha 3 subunit isoform 2
"""synovial sarcoma, translocated to X chromosome"""
"""cadherin 2, type 1 preproprotein"""
desmocollin 3 isoform Dsc3a preproprotein
chromosome 18 open reading frame 34
dystrobrevin alpha isoform 5
chromosome 18 open reading frame 21
"""phosphoinositide-3-kinase, class 3"""
SET binding protein 1
proline-serine-threonine phosphatase interacting
"""MAD, mothers against decapentaplegic homolog 2"""
ribosomal protein L17
maestro
deleted in colorectal carcinoma
methyl-CpG binding domain protein 2 isoform 1
thioredoxin-like 1
ubiquitin-protein ligase NEDD4-like
KIAA1983 protein
"""cadherin 20, type 2 preproprotein"""
B-cell lymphoma protein 2 alpha isoform
"""cadherin 7, type 2 preproprotein"""
NCAG1
chromosome 18 open reading frame 14
suppressor of cytokine signaling 6
cerebellin 2 precursor
F-box only protein 15
FLJ44313 protein
sal-like 3

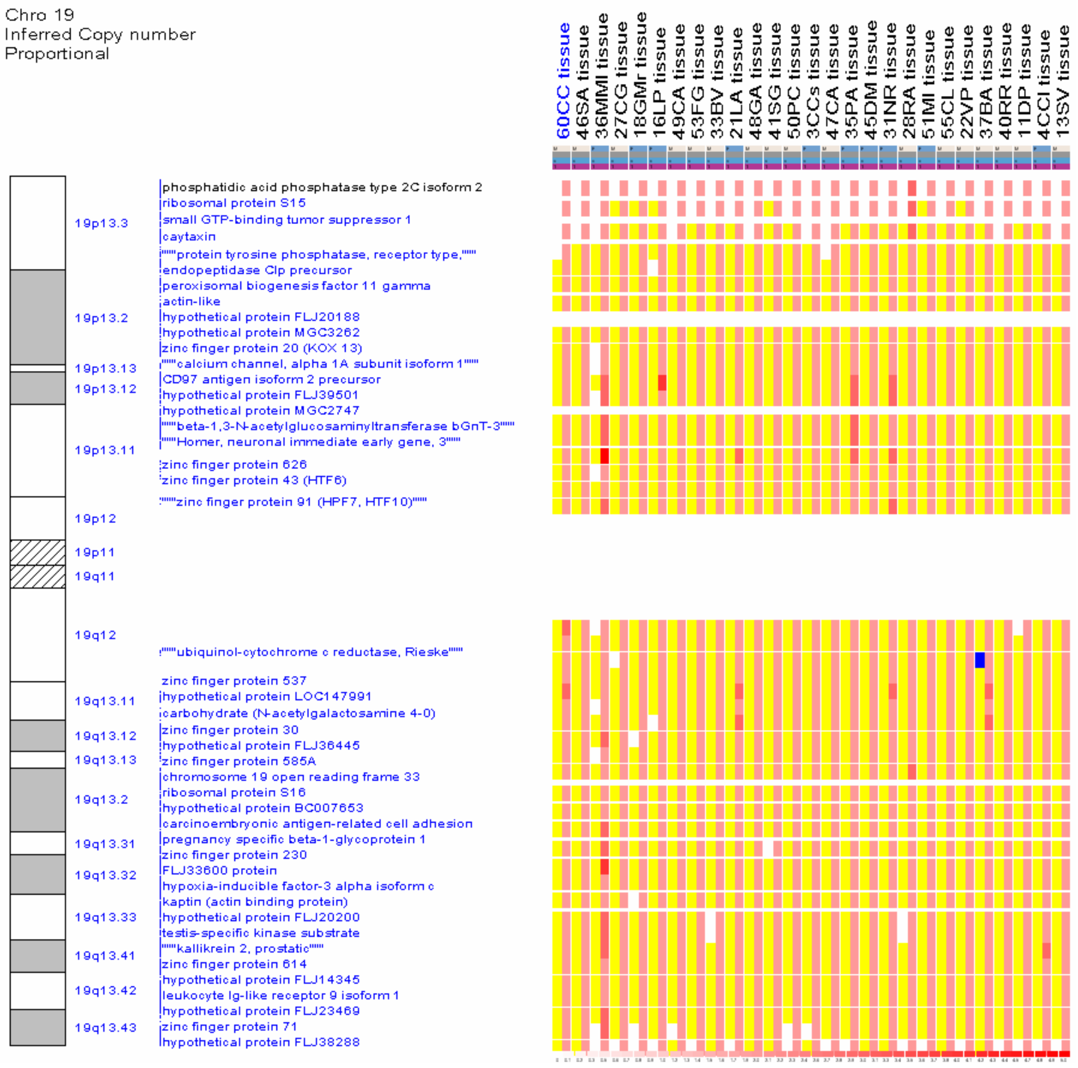
Chro 19
Inferred Copy number
Proportional
60CC tissue
46SA tissue
36MMI tissue
27CG tissue
18GMr tissue
16LP tissue
49CA tissue
53FG tissue
33BV tissue
21LA tissue
48GA tissue
41SG tissue
50PC tissue
3CCs tissue
47CA tissue
35PA tissue
45DM tissue
31NR tissue
28RA tissue
51MI tissue
55CL tissue
22VP tissue
37BA tissue
40RR tissue
11DP tissue
4CCI tissue
13SV tissue
19p13.3
19p13.2
19p13.13
19p13.12
19p13.11
19p12
19p11
19q11
19q12
19q13.11
19q13.12
19q13.13
19q13.2
19q13.31
19q13.32
19q13.33
19q13.41
19q13.42
19q13.43
phosphatidic acid phosphatase type 2C isoform 2
ribosomal protein S15
small GTP-binding tumor suppressor 1
caytaxin
"""protein tyrosine phosphatase, receptor type,"""
endopeptidase Clp precursor
peroxisomal biogenesis factor 11 gamma
actin-like
hypothetical protein FLJ20188
hypothetical protein MGC3262
zinc finger protein 20 (KOX 13)
"""calcium channel, alpha 1A subunit isoform 1"""
CD97 antigen isoform 2 precursor
hypothetical protein FLJ39501
hypothetical protein MGC2747
"""beta-1,3-N-acetylglucosaminyltransferase bGnT-3"""
"""Homer, neuronal immediate early gene, 3"""
zinc finger protein 626
zinc finger protein 43 (HTF6)
"""zinc finger protein 91 (HPF7, HTF10)"""
"""ubiquinol-cytochrome c reductase, Rieske"""
zinc finger protein 537
hypothetical protein LOC147991
carbohydrate (N-acetylgalactosamine 4-0)
zinc finger protein 30
hypothetical protein FLJ36445
zinc finger protein 585A
chromosome 19 open reading frame 33
ribosomal protein S16
hypothetical protein BC007653
carcinoembryonic antigen-related cell adhesion
pregnancy specific beta-1-glycoprotein 1
zinc finger protein 230
FLJ33600 protein
hypoxia-inducible factor-3 alpha isoform c
kaptin (actin binding protein)
hypothetical protein FLJ20200
testis-specific kinase substrate
"""kallikrein 2, prostatic"""
zinc finger protein 614
hypothetical protein FLJ14345
leukocyte Ig-like receptor 9 isoform 1
hypothetical protein FLJ23469
zinc finger protein 71
hypothetical protein FLJ38288

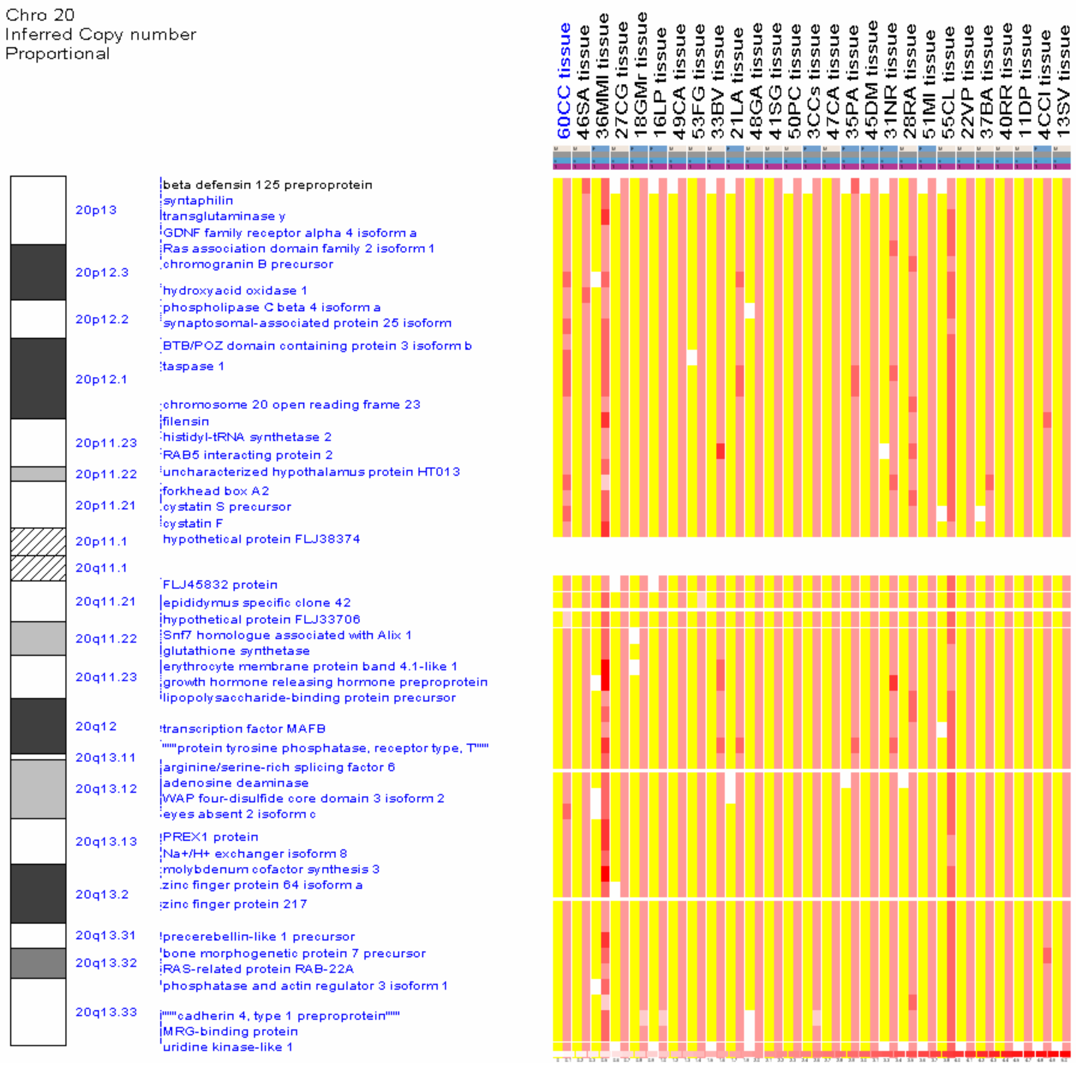

Chro 20
Inferred Copy number
Proportional
60CC tissue
46SA tissue
36MMI tissue
27CG tissue
18GMr tissue
16LP tissue
49CA tissue
53FG tissue
33BV tissue
21LA tissue
48GA tissue
41SG tissue
50PC tissue
3CCs tissue
47CA tissue
35PA tissue
45DM tissue
31NR tissue
28RA tissue
51MI tissue
55CL tissue
22VP tissue
37BA tissue
40RR tissue
11DP tissue
4CCI tissue
13SV tissue
20p13
20p12.3
20p12.2
20p12.1
20p11.23
20p11.22
20p11.21
20p11.1
20q11.1
20q11.21
20q11.22
20q11.23
20q12
20q13.11
20q13.12
20q13.13
20q13.2
20q13.31
20q13.32
20q13.33
beta defensin 125 preproprotein
syntaphilin
transglutaminase y
GDNF family receptor alpha 4 isoform a
Ras association domain family 2 isoform 1
chromogranin B precursor
hydroxyacid oxidase 1
phospholipase C beta 4 isoform a
synaptosomal-associated protein 25 isoform
BTB/POZ domain containing protein 3 isoform b
taspase 1
chromosome 20 open reading frame 23
filensin
histidyl-tRNA synthetase 2
RAB5 interacting protein 2
uncharacterized hypothalamus protein HT013
forkhead box A2
cystatin S precursor
cystatin F
hypothetical protein FLJ38374
FLJ45832 protein
epididymus specific clone 42
hypothetical protein FLJ33706
Snf7 homologue associated with Alix 1
glutathione synthetase
erythrocyte membrane protein band 4.1-like 1
growth hormone releasing hormone preproprotein
lipopolysaccharide-binding protein precursor
transcription factor MAFB
"""protein tyrosine phosphatase, receptor type, T"""
arginine/serine-rich splicing factor 6
adenosine deaminase
WAP four-disulfide core domain 3 isoform 2
eyes absent 2 isoform c
PREX1 protein
Na+/H+ exchanger isoform 8
molybdenum cofactor synthesis 3
zinc finger protein 64 isoform a
zinc finger protein 217
precerebellin-like 1 precursor
bone morphogenetic protein 7 precursor
RAS-related protein RAB-22A
phosphatase and actin regulator 3 isoform 1
"""cadherin 4, type 1 preproprotein"""
MRG-binding protein
uridine kinase-like 1

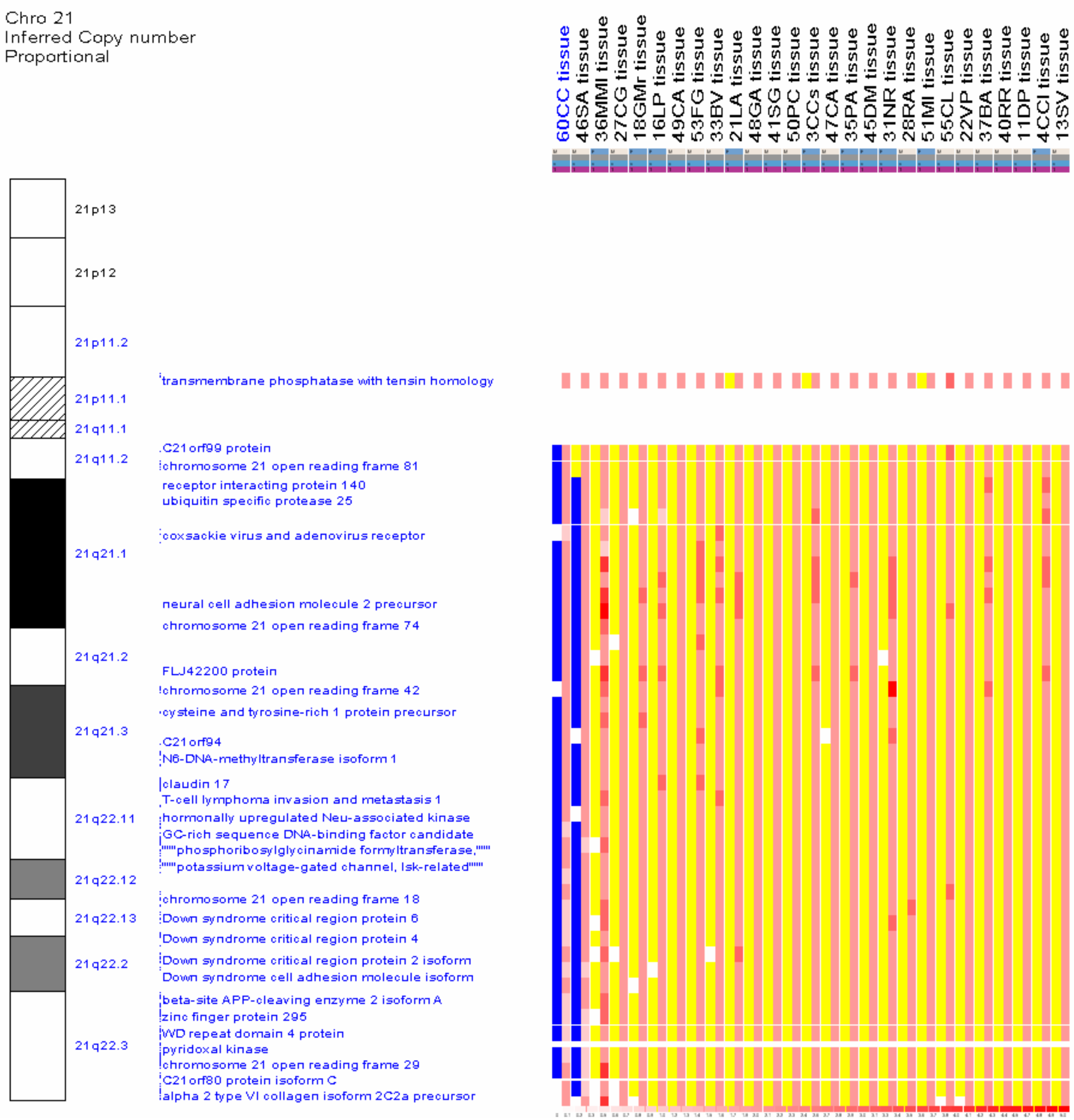

Chro 21
Inferred Copy number
Proportional
60CC tissue
46SA tissue
36MMI tissue
27CG tissue
18GMr tissue
16LP tissue
49CA tissue
53FG tissue
33BV tissue
21LA tissue
48GA tissue
41SG tissue
50PC tissue
3CCs tissue
47CA tissue
35PA tissue
45DM tissue
31NR tissue
28RA tissue
51MI tissue
55CL tissue
22VP tissue
37BA tissue
40RR tissue
11DP tissue
4CCI tissue
13SV tissue
21p13
21p12
21p11.2
21p11.1
21q11.1
21q11.2
21q21.1
21q21.2
21q21.3
21q22.11
21q22.12
21q22.13
21q22.2
21q22.3
transmembrane phosphatase with tensin homology
C21orf99 protein
chromosome 21 open reading frame 81
receptor interacting protein 140
ubiquitin specific protease 25
coxsackie virus and adenovirus receptor
neural cell adhesion molecule 2 precursor
chromosome 21 open reading frame 74
FLJ42200 protein
chromosome 21 open reading frame 42
cysteine and tyrosine-rich 1 protein precursor
C21orf94
N6-DNA-methyltransferase isoform 1
claudin 17
T-cell lymphoma invasion and metastasis 1
hormonally upregulated Neu-associated kinase
GC-rich sequence DNA-binding factor candidate
""""phosphoribosylglycinamide formyltransferase,""""
""""potassium voltage-gated channel, Isk-related""""
chromosome 21 open reading frame 18
Down syndrome critical region protein 6
Down syndrome critical region protein 4
Down syndrome critical region protein 2 isoform
Down syndrome cell adhesion molecule isoform
beta-site APP-cleaving enzyme 2 isoform A
zinc finger protein 295
WD repeat domain 4 protein
pyridoxal kinase
chromosome 21 open reading frame 29
C21orf80 protein isoform C
alpha 2 type VI collagen isoform 2C2a precursor

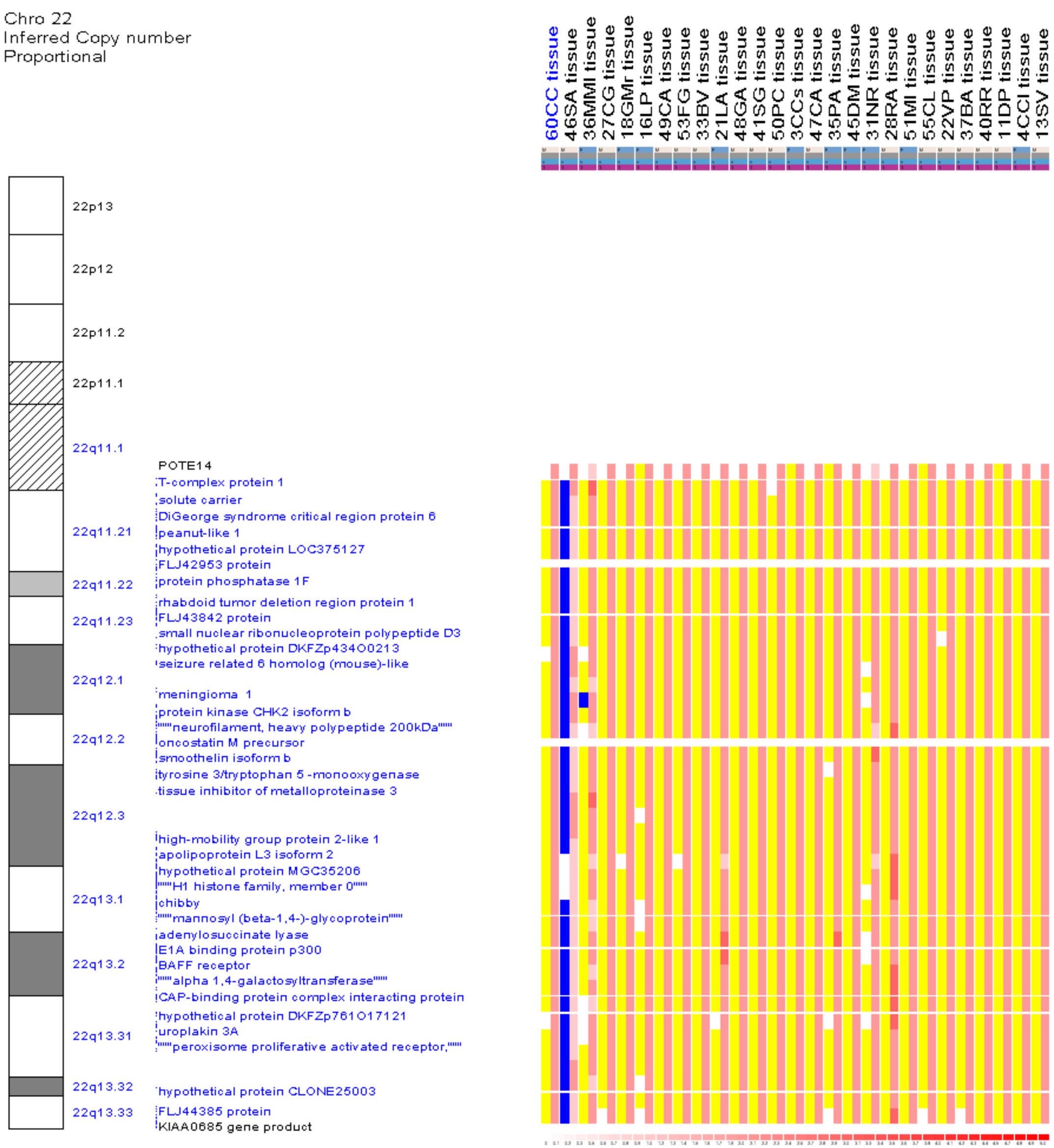
Chro 22
Inferred Copy number
Proportional
60CC tissue
46SA tissue
36MMI tissue
27CG tissue
18GMr tissue
16LP tissue
49CA tissue
53FG tissue
33BV tissue
21LA tissue
48GA tissue
41SG tissue
50PC tissue
3CCs tissue
47CA tissue
35PA tissue
45DM tissue
31NR tissue
28RA tissue
51MI tissue
55CL tissue
22VP tissue
37BA tissue
40RR tissue
11DP tissue
4CCI tissue
13SV tissue
22p13
22p12
22p11.2
22p11.1
22q11.1
22q11.21
22q11.22
22q11.23
22q12.1
22q12.2
22q12.3
22q13.1
22q13.2
22q13.31
22q13.32
22q13.33
POTE14
T-complex protein 1
solute carrier
DiGeorge syndrome critical region protein 6
peanut-like 1
hypothetical protein LOC375127
FLJ42953 protein
protein phosphatase 1F
rhabdoid tumor deletion region protein 1
FLJ43842 protein
small nuclear ribonucleoprotein polypeptide D3
hypothetical protein DKFZp434O0213
seizure related 6 homolog (mouse)-like
meningioma 1
protein kinase CHK2 isoform b
""""neurofilament, heavy polypeptide 200kDa""""
oncostatin M precursor
smoothelin isoform b
tyrosine 3/tryptophan 5 -monooxygenase
tissue inhibitor of metalloproteinase 3
high-mobility group protein 2-like 1
apolipoprotein L3 isoform 2
hypothetical protein MGC35206
""""H1 histone family, member 0""""
chibby
""""mannosyl (beta-1,4-)-glycoprotein""""
adenylosuccinate lyase
E1A binding protein p300
BAFF receptor
""""alpha 1,4-galactosyltransferase""""
CAP-binding protein complex interacting protein
hypothetical protein DKFZp761O17121
uroplakin 3A
""""peroxisome proliferative activated receptor,""""
hypothetical protein CLONE25003
FLJ44385 protein
KIAA0685 gene product
