# Supplementary material for: Genome-wide screening of copy number alterations and LOH events in renal cell carcinomas and integration with gene expression profile
Source: Mol Cancer. 2008 Jan 14;7:6. doi: 10.1186/1476-4598-7-6 (PMC2253555; doi:10.1186/1476-4598-7-6)
Supplement: Additional file 5 — Clear cell renal carcinoma samples analyzed in this study and corresponding clinical data. Cases are listed in order of increasing tumor size, stage and grade classes. For each case, patient's gender, tumor size at the time of surgery, tumor stage and nuclear grade classifications and time elapsed from surgery are reported. These samples were analyzed for genomic (on GeneChip 100K SNP mapping arrays) or transcriptomic (on HG-U133 Plus 2.0 arrays) profiles. *All patients were scheduled for total removal of affected kidney, except for two which underwent partial nephrectomy. **Two patients presented tumor recurrence. Abbreviations: M, male; F, female; NA, data not available. [file 1476-4598-7-6-S5.pdf]

Sample No.	Gender	Tumor diameter (cm)	Tumor stage pT	Nuclear grade G	Elapsed time (months)	100K SNP array	HG-U133 Plus 2.0 array
13SV	M	1.5	1	1	55	x	
4CCl	F	2.0	1	1	63	x	
52CA	F	0.8	2	2	23		x
11DP	M	2.1 *	1	2	56	x	
44DE	F	2.5	1	2	27		x
40RR	M	2.7	1	2	30	x	x
37BA	M	3.4	1	2	30	x	x
22VP	M	0.1	1	2	49	x	
55CL	M	3.2	1	3	20	x	
51MI	F	4.2	1	2	24	x	x
28RA	M	4.5	NA	2	40	x	x
31NR	F	4.5	1	2	35	x	x
45DM	F	4.5	1	2	26	x	x
35PA	F	4.0 *	NA	3	31 **	x	
47CA	M	5.0	1	2	25	x	x
3CCs	F	5.5 *	1	2	63	x	
50PC	M	5.7	1	2	24	x	x
41SG	M	6.5	1	2	30	x	
48GA	M	6.5	1	2	25	x	
21LA	F	7.0	1	2	50	x	
33BV	M	5.2	3	2	33	x	x
53FG	M	6.5	1	2	21	x	
49CA	M	6.5	3	3	25	x	x
32GM	F	8.5	2	2	35		x
16LP	F	8.0	3	2	53	x	
18GMr	F	8.0	3	2	51	x	
27CG	M	8.0	3	2	44	x	x
36MMl	F	9.5	2	2	31	x	x
46SA	F	10.0	2	2	26	x	x
60CC	M	11.5	3	3	18 **	x	
