# Supplementary material for: Genome-wide screening of copy number alterations and LOH events in renal cell carcinomas and integration with gene expression profile
Source: Mol Cancer. 2008 Jan 14;7:6. doi: 10.1186/1476-4598-7-6 (PMC2253555; doi:10.1186/1476-4598-7-6)
Supplement: Additional file 6 — Complete description about methods used to prepare biological samples for microarray experiments and about statistical procedures implemented to analyse data. [file 1476-4598-7-6-S6.pdf]

Complete description about methods and statistical procedures

Sample preparation for Affymetrix GeneChip® analysis

Genomic DNA from the 27 tumor tissues and corresponding blood samples was prepared for whole-genome SNP mapping using both GeneChip® Human Mapping 50K Xba and 50K Hind assay kits (Affymetrix, Santa Clara, CA, USA) according to the manufacturer's protocols. Briefly, 250 ng genomic DNA was separately digested with *Xba*I and *Hind*III restriction enzymes, ligated to synthetic adapters and amplified by PCR using a universal primer. After the presence of the typical PCR banding pattern was confirmed by means of microcapillary electrophoresis, 40 μg PCR product was fragmented with DNase I, end-labelled with biotin and hybridized for 17 h at 48°C onto GeneChip® Human Mapping 50K Xba and 50K Hind arrays (Affymetrix). Arrays were washed and stained on a Fluidics Station 450 (Affymetrix) according to the manufacturer's instructions. Fluorescent signals corresponding to each SNP were acquired using GeneChip® Scanner 3000 and quantified by GTYPE software v4.0 (Affymetrix). The combination of the two GeneChip® Human Mapping arrays, referred to as 100K SNP mapping array set, allowed the genotyping of 116,204 SNPs distributed over all chromosomes, excluding the Y chromosome.

Total RNA from tumor tissues and healthy cortical tissues (16 and 11 samples, respectively) was prepared using GeneChip® Two Cycle cDNA Synthesis kit and GeneChip® IVT Labeling kit (Affymetrix), according to the manufacturer's protocols. Briefly, starting from 40 ng total RNA, biotinylated cRNA was produced by two subsequent cycles of reverse transcription and in vitro transcription (IVT). Biotinylated cRNA (15 μg) was fragmented and hybridized for 16 h at 45°C onto GeneChip® Human Genome U133 Plus 2.0 arrays (Affymetrix). After washing and staining, fluorescent microarray images were acquired and analyzed with GCOS software (Affymetrix). All GeneChip® data files are available at ArrayExpress repository (E-TABM-282, E-TABM-283 and E-TABM-284).

Assessment of genomic copy number alterations and LOH events in RCC samples

Genomic regions with copy number alterations (CNAs) were first assessed using Copy Number Analyzer for GeneChip software (CNAG, v2.0)[1]. Using the "Integral view" option, we compared the 27 tumor samples to the corresponding blood samples and displayed in a unique plot all gains and losses occurring along each chromosome. Datasets from only GeneChip® Human Mapping 50K Hind arrays were used. Briefly, CNAG imported CEL files from GTYPE software (Affymetrix) and converted probeset signal intensities into SNP copy number values. Using default parameters, CNAG averaged these copy number values over windows of 10 contiguous SNPs after having first reduced noise by discarding data for the two SNPs with the highest and lowest copy number values in each window. Using the locally averaged data, the resulting map was visually inspected to identify chromosomal regions affected by DNA alterations (copy number gains and losses). Data regarding the X chromosome were excluded.

Copy number alterations were also assessed using dChip2006 software[2], which offers the possibility of combining the two datasets from GeneChip® Human Mapping 50K Xba and 50K Hind arrays. dChip2006 was used to calculate a raw copy number value for each SNP in tumor and normal samples. Then, we implemented a home-made procedure to calculate aberrant regions. Specifically, single SNP copy number values were smoothed over contiguous "nodes" of 1 Mb, giving a single copy number value for each node; this calculation was performed in R using the *lokern* package (http://www.r-project.org). We decided that a copy number (CN) value more than 2.0 defined CN gain, while a value less than 1.8 defined CN loss. For the purpose of this study, regions of CN gain were defined as two or more consecutive nodes with copy number gain in at least 11 tumor samples, while regions of CN loss were defined as two or more consecutive nodes with copy number loss.

---

[1] Nannya Y, Sanada M, Nakazaki K *et al.* A robust algorithm for copy number detection using high-density oligonucleotide single nucleotide polymorphism genotyping arrays. *Cancer Res* 2005; **65**: 6071-6079.

[2] Lin M, Wei LJ, Sellers WR *et al.* dChipSNP: significance curve and clustering of SNP-array-based loss-of-heterozygosity data. *Bioinformatics* 2004; **20**: 1233-1240.

dChip2006 was also used to identify LOH events (SNPs changing from a heterozygous genotype in a normal sample to a homozygous one in the matched tumor sample) and to define a summary LOH score for each SNP taking into account all the 27 sample pairs. Then, applying a home-made procedure, LOH scores of single SNPs were grouped into contiguous nodes of 1 Mb in length, and the maximum LOH score was chosen for each node. To identify regions affected by statistically significant LOH, different LOH score thresholds were examined to find a value that balanced stringency and noise. In addition, the CN status of these regions was evaluated to distinguish LOH events with and without CN changes.

Using the UCSC Genome Browser database (http://genome.ucsc.edu), all genes located in aberrant regions were identified. Genes located in LOH regions with CN gain were assigned to the "LOH+CN gain" class, while those in LOH regions with both CN loss and neutral status in different sanples were assigned to the "LOH+CN loss" class to avoid redundancy in the final gene list. Literature mining was carried out with MILANO tool (Microarray Literature-based Annotation; http://milano.md.huji.ac.il/), using the cancer-related keywords: oncogene, tumor suppressor, secreted, metastasis, progression, prognosis, amplification, deletion, LOH, upregulation, downregulation, methylation. All procedures were implemented using in-house Python scripts.

Transcriptomic profiling and integration with copy number data

Differential gene expression analysis was performed comparing the 16 RCC tissue samples to the 11 normal cortical samples. Briefly, GeneChip® HG-U133 Plus 2.0 array probesets were annotated using an updated custom chip description file [3] (available at http://brainarray.mbni.med.umich.edu/CustomCDF), thus obtaining 17,663 unique Entrez Gene IDs and corresponding chromosomal positions. Raw signal intensities were converted into expression values using the robust multi-array average (RMA) procedure, and differentially expressed genes

[3] Dai M, Wang P, Boyd AD *et al*. Evolving gene/transcript definitions significantly alter the interpretation of GeneChip data. *Nucleic Acids Res* 2005; **33**: e175.

(DEGs) were identified by the t statistic of the significance analysis of microarrays (SAM), using the package *samr* (v1.8; http://www-stat.stanford.edu/~tibs/SAM/) and setting 90th percentile FDR=0 and q-value=0. Functional annotation of the DEG list was carried out using DAVID (Database for Annotation, Visualization and Integrated Discovery, http://david.abcc.ncifcrf.gov), with default parameters (EASE threshold 0.1, minimal count 2). The Affymetrix GeneChip HG-U133 Plus 2.0 array was chosen as default background for enrichment analysis.

Furthermore, we compared our DEG list with the microarray gene expression dataset concerning RCC samples published by Jones *et al*. and including differential genes that overlap with the dataset provided by Lenburg *et al*. [4] (available at http://www.bidmcgenomics.org/KidneyCancer/index.html). The DEGs found in common were functionally annotated with DAVID as described earlier, but using our complete DEG list as background for the enrichment analysis.

Finally, the list of genes in chromosomal regions with significant CNAs was compared to our DEG list to investigate the expression levels of genes in aberrant regions. After dividing these DEGs according to their distribution into regions of CN gain and CN “non-gain” (i.e. significantly affected by deletions or LOH), their RMA expression values calculated for each tumor and normal sample were plotted in a heat map using the TIGR Multiple Experiment Viewer (TMEV v4.0, http://www.tm4.org/mev.html). The software converted each RMA value into a color on a gradient from green to red. Default parameters were used except for the color scale range, which was set from 3.75 as lower limit to 9.33 as upper limit.

[4] Jones J, Out H, Spentzos D *et al*. Gene signatures of progression and metastasis in renal cell cancer. *Clin Cancer Res* 2005; **11**: 5730-5739.
